# Solvent Deuterium Isotope Effects of Substrate Reduction by Nitrogenase from *Azotobacter vinelandii*

**DOI:** 10.1021/jacs.2c07574

**Published:** 2022-11-08

**Authors:** Siobhán G. MacArdle, Douglas C. Rees

**Affiliations:** Howard Hughes Medical Institute, Department of Chemistry and Chemical Engineering, California Institute of Technology, Pasadena, California91125, United States

## Abstract

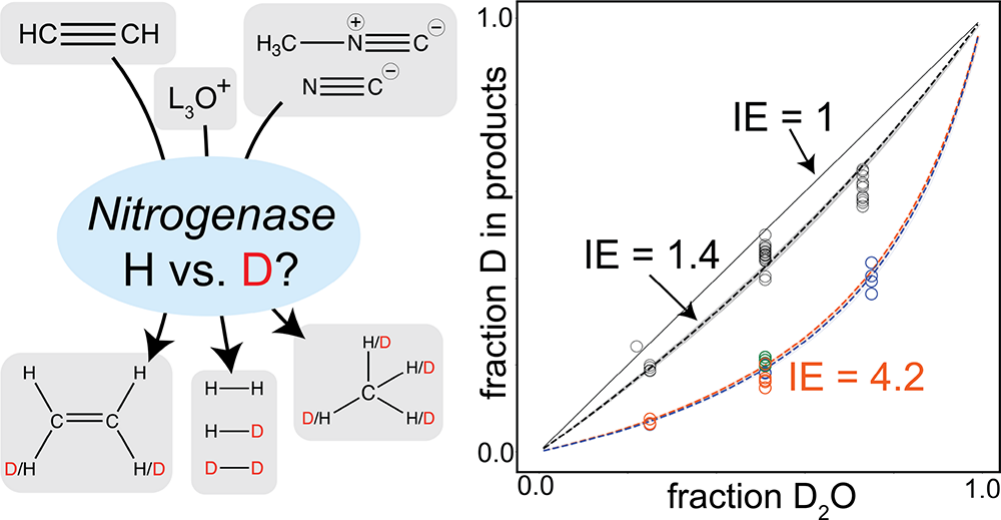

The mechanism of nitrogenase, the enzyme responsible for biological nitrogen fixation, has been of great interest for understanding the catalytic strategy utilized to reduce dinitrogen to ammonia under ambient temperatures and pressures. The reduction mechanism of nitrogenase is generally envisioned as involving multiple cycles of electron and proton transfers, with the known substrates requiring at least two cycles. Solvent kinetic isotope effect experiments, in which changes of reaction rates or product distribution are measured upon enrichment of solvent with heavy atom isotopes, have been valuable for deciphering the mechanism of complex enzymatic reactions involving proton or hydrogen transfer. We report the distribution of ethylene, dihydrogen, and methane isotopologue products measured from nitrogenase-catalyzed reductions of acetylene, protons, and cyanide, respectively, performed in varying levels of deuterium enrichment of the solvent. As has been noted previously, the total rate of product formation by nitrogenase is largely insensitive to the presence of D_2_O in the solvent. Nevertheless, the incorporation of H/D into products can be measured for these substrates that reflect solvent isotope effects on hydrogen atom transfers that are faster than the overall rate-determining step for nitrogenase. From these data, a minimal isotope effect is observed for acetylene reduction (1.4 ± 0.05), while the isotope effects for hydrogen and methane evolution are significantly higher at 4.2 ± 0.1 and 4.4 ± 0.1, respectively. These results indicate that there are pronounced differences in the sensitivity to isotopic substitution of the hydrogen atom transfer steps associated with the reduction of these substrates by nitrogenase.

## Introduction

Nitrogenase is the only known enzyme that can convert atmospheric dinitrogen into ammonia, an integral building block for the many types of biomolecules found in all organisms.^1^ The focus of this study, the molybdenum (Mo)-nitrogenase from the free-living soil bacteria *Azotobacter vinelandii*, is the default nitrogenase for this organism, although vanadium and iron-containing forms may be expressed under appropriate limiting conditions.^2,3^ Nitrogenase consists of two component proteins, the molybdenum iron (MoFe) protein that houses the active site and the iron (Fe) protein that mediates the ATP-dependent transfer of electrons to the MoFe protein, among other activities (reviewed in Burgess and Lowe,^4^ Seefeldt et al.,^5^ and Einsle and Rees^6^). The MoFe protein contains two complex metalloclusters: the active site FeMo-cofactor, a [Fe_7_S_9_Mo] cluster with an interstitial carbide and a coordinating *R*-homocitrate, and the [Fe_8_S_7_] P-cluster involved in electron transfer from the [Fe_4_S_4_] cluster of the Fe protein.

Decades of study on nitrogenase have revealed detailed information about the structure and activity of the enzyme; however, the complex mechanism of substrate reduction remains incomplete. Although the reduction of N_2_ to NH_3_ is thermodynamically favorable under standard conditions, there is a large kinetic barrier for the reaction that is associated with a significant energy requirement both for the biological and industrial processes. In addition to the 8 electrons required to reduce N_2_ to NH_3_ when coupled to obligatory hydrogen evolution,^7^ the nitrogenase-catalyzed reaction also requires at least 16 MgATP per N_2_ fixed, so that the overall reaction stoichiometry is typically formulated as
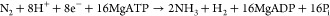


In addition to dinitrogen, nitrogenase can reduce a variety of other substrates including C_2_H_2_, HCN, CH_3_NC, SCN^–^, SeCN^–^, and N^3–^. In the absence of other substrates, or at sufficiently low concentrations of other substrates, nitrogenase will also reduce protons to form H_2_.

Reducing equivalents are transferred to the active site of the MoFe protein from the [Fe_4_S_4_] cluster of the reduced Fe protein through a cycle of protein–protein association, ATP hydrolysis coupled to intermolecular electron transfer, and the subsequent dissociation of the Fe protein–MoFe protein complex.^8,9^ Each of these Fe protein cycles of binding, ATP hydrolysis, and electron transfer are responsible for the ultimate transfer of one electron to the active site. An important aspect of the nitrogenase reaction is that dinitrogen does not bind to the resting state of the enzyme; instead, the active site FeMo-cofactor must be reduced by 3–4 electrons before N_2_ can bind,^10,11^ coupled to the reductive elimination of H_2_.^12^ Subsequent cycles of electron transfer culminate in the reduction to ammonia. Although dinitrogen can only bind to the enzyme after 3–4 rounds of electron transfer, other substrates can bind to less-reduced states of the enzyme. Acetylene, methyl isocyanide, and cyanide, substrates investigated in this work, can bind to the enzyme after only one round of electron transfer from the Fe protein,^13−15^ and there is evidence indicating that these substrates may bind to the resting state.^16−19^ Dihydrogen evolution, the other process investigated here, can occur after two rounds of electron transfer to the active site. Despite significant efforts (reviewed in Seefeldt et al.^5^), mechanistic details of the pathway for substrate reduction remain incomplete. Here, we report the use of solvent isotope experiments to provide information about the proton transfer steps of the nitrogenase reduction process.

Stable isotope substitutions, the focus of this report, have a rich history of providing information about how nitrogenase reacts with substrates. One of the most important discoveries of the biological nitrogen fixation reaction, that ammonia is the product of nitrogenase, was established by Burris and co-workers using ^15^N_2_ as the substrate for *Azotobacter agile* and detecting ^15^N-labeled ammonia.^20^ Another heavy isotope, deuterium, was paramount to discovering the important relationship between H_2_ and N_2_ during nitrogen fixation. In N_2_ fixing conditions under D_2_ (in H_2_O), nitrogenase will produce HD; formation of the HD product is largely dependent on the presence of N_2_ and consumes two electrons per HD formed.^21,22^ This observation was groundbreaking and inspired much future work toward elucidating the role of H_2_ evolution in N_2_ reduction.

Stable isotopes have further provided information about the orientation, stoichiometry, and exchange properties of acetylene binding to the nitrogenase active site. Acetylene reduction assays with C_2_D_2_ (in H_2_O) provided the valuable insight that nitrogenase predominantly produces *cis*-C_2_H_2_D_2_ with only a small fraction of the *trans*-species.^23^ Using ^13^C-acetylene and ^13^C ENDOR, Lee et al. were able to detect two acetylene molecules bound to the FeMo-cofactor of a nitrogenase mutant, while ^1^H ENDOR on the C_2_H_2_- and C_2_D_2_-bound species in H_2_O and D_2_O established that H/D atoms on the bound acetylene cannot exchange with solvent.^24^ The methane products of CH_3_NC reduction by nitrogenase in D_2_O appeared as CD_4_, indicating that the carbon in the products arose from the isocyanide carbon of the substrate.^25^ In addition to these qualitative observations, stable isotopes were used to quantitatively measure the ^15^N/^14^N kinetic isotope effect for N_2_ reduction using isotope ratio mass spectrometry (1.017 ± 0.002).^26^

The deuterium isotope is particularly valuable for mechanistic explorations because the factor of two difference in atomic mass between H and D contributes to a potentially significant kinetic isotope effect. A kinetic isotope effect is defined as a change in the rate of a reaction upon isotopic substitution of atoms in the reaction. These effects can provide information about the atoms involved in the reaction mechanism, the structure of the transition state, and the rate-limiting step. Solvent isotope experiments are a type of isotope study in which the protons of the solvent are replaced with deuterium (in this case, H_2_O replaced with D_2_O).^27^ For a change in rate to be observed upon deuterium enrichment of the solvent, solvent H/D atoms must be involved in the rate-limiting step. The rate-limiting step in the nitrogenase mechanism has been identified as the dissociation of inorganic phosphate from the Fe protein after ATP hydrolysis during the electron transfer cycle,^28^ of which multiple cycles are necessary to build up reducing equivalents at the active site of the MoFe protein. With the exception of the reduction of acrylonitrile to propane and propylene,^29^ changes in the rate of product formation upon D_2_O enrichment of the solvent have not been observed with nitrogenase. This behavior is consistent with any changes in rate associated with H^+^/D^+^ transfer at the active site being eclipsed by the slower dissociation of P_i_ from the Fe protein in each electron transfer cycle. However, the insensitivity of the rate of product formation to isotopic substitution does not preclude the use of solvent isotope experiments to learn about the nitrogenase mechanism. For irreversible reactions that result in incorporation of nonexchangeable H atoms into products (such as ethylene, dihydrogen, and methane production by nitrogenase), the relative amount of deuterium incorporation into the products can be used to unmask the isotope effects associated with hydrogen transfer at the active site and can shed light on the reaction pathway. These measurements are often termed product deuterium isotope effect experiments.^30^

Isotope effect experiments in mixed H_2_O/D_2_O buffers offer advantages over those determined from separate assays in either H_2_O or D_2_O because errors due to differences in experimental conditions such as pL (L denotes either H or D) and temperature across reaction vials can be minimized, and there is no need to correct for secondary solvent isotope effects.^31^ When the reaction mechanism involves the transfer of one hydrogen, the isotope effect is simply the ratio of the amount of products with hydrogen incorporated to the amount of products with deuterium incorporated divided by the H_2_O/D_2_O ratio of the solvent.^31,32^ When more than one hydrogen transfer occurs, as is the case with all known reductions performed by nitrogenase, products with multiple levels of deuteration can be formed, and the isotope effect can be calculated from the relative levels of these products.

**Scheme 1:**
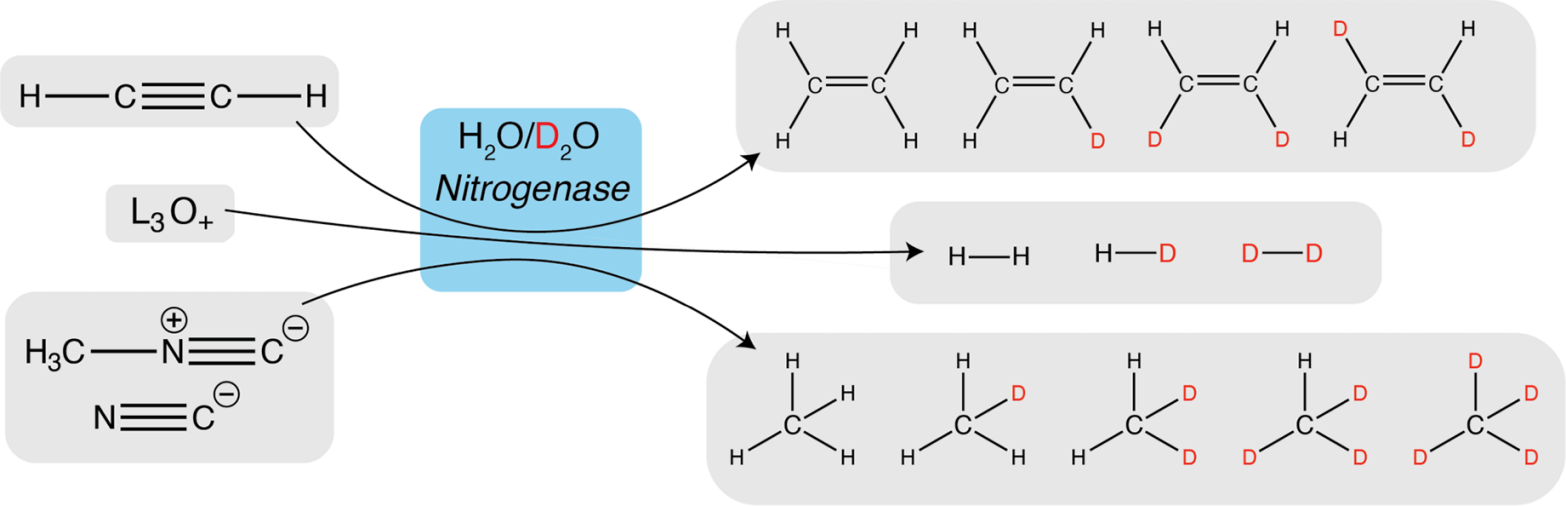
Isotope Effect Experiments of Acetylene, Cyanide, Methyl Isocyanide and Proton Reduction Assays by Nitrogenase from *A. vinelandii* Reduction assays were performed in mixed H_2_O/D_2_O buffers, and the relative amounts of product isotopologues were quantified. Products of acetylene reduction assays are *d*_0_-*ethylene*, *d*_1_-*ethylene*, *cis*-*d*_2_-*ethylene,* and *trans*-*d*_2_-*ethylene*, the latter a minor species. Products of proton reduction assay are H_2_, HD, and D_2_. Products of cyanide and methyl isocyanide reduction assays are *d*_0_-*methane*, *d*_1_-*methane*, *d*_2_-*methane*, *d*_3_-*methane*, and *d*_4_-*methane*. All nitrogenase assays require the ATP regeneration system and a reductant (in this case, dithionite).

In this study, we evaluated the potential role of solvent isotope effects in the nitrogenase mechanism by performing acetylene, proton, and cyanide reduction assays in buffers of varying mole fraction of deuterium (Scheme 1). The products of these reactions, ethylene, dihydrogen, and methane, respectively, are well suited for these studies since the H/D(s) incorporated during substrate reduction do not subsequently exchange with solvent, a process that precludes characterization of the ammonia product of dinitrogen reduction by this approach. The product isotopologues were quantified by GC–MS, FTIR, and ^1^H NMR, and these data were used to determine the isotope effect associated with the reduction of acetylene to ethylene, protons to H_2_, and cyanide to methane. Interestingly, these results show that the isotope effect of acetylene reduction by nitrogenase is small (1.4 ± 0.05) while those of the reduction of protons to H_2_ and cyanide to methane are significantly greater at 4.2 ± 0.1 and 4.4 ± 0.1, respectively. These results demonstrate that the details of the H atom transfer steps associated with acetylene reduction differ from those associated with cyanide and proton reduction. We note that our results from proton reduction assays are comparable to an early report that quantified dihydrogen isotopologues produced by partially purified nitrogenase from *A. vinelandii*.^33^

Since the rate of electron transfer through nitrogenase is generally independent of the substrate reduced, nitrogenase may be considered to be a relatively nonspecific, sequential deliverer of electrons and protons to substrates.^4^ The ability of nitrogenase to serve as a hydrogenation catalyst has been captured in the mechanistic paradigm that “substrate and H atoms are bound contiguously in the reaction zone, and H atoms are transferred to the substrate to form product”.^34^ In the simplest case, there could be a universal mechanism of H atom transfer, such that the level of H/D isotope discrimination during reduction would be independent of the substrate. However, the significantly different solvent isotope effects that we observe in the reduction of acetylene compared to cyanide, isocyanide, and proton reduction support variations in the H atom transfer steps during reduction of these substrates and are indicative of differences in the detailed reduction mechanisms of different substrates by nitrogenase.

## Experimental Section

### Materials and Buffer Preparation

Dithionite and all components of the ATP regeneration system (creatine phosphate, ATP, creatine phosphokinase) were purchased from Sigma-Aldrich. Tris buffer (50 mM, pL = 7.8) was prepared in H_2_O and D_2_O (Cambridge Isotope Labs) and combined in appropriate ratio for the desired final D_2_O composition of assay. Buffers were adjusted to pL = 7.8 with either HCl or DCl. For buffers containing D_2_O, the pH meter was first immersed in 100% D_2_O for 20 min prior to use. The following calculation was used to convert the reading on the pH meter (pH_obs_) to the actual pL of the solution^35^
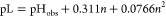
where *n* is the mole fraction of deuterium in the solution. The mole fraction of deuterium in the final buffers was confirmed by ^1^H NMR, and the error in D_2_O percentage of the solution was measured to be 3%.

### Nitrogenase Acetylene Reduction Assay

Nitrogenase from *A. vinelandii* was purified as previously described (see Supporting Information for representative chromatograms and denaturing gels).^36^ The ATP regeneration system (final concentrations: 15 mM creatine phosphate, 3.8 mM ATP, 3.8 mM MgCl_2_, 50 units/mL creatine phosphokinase) was prepared in buffer (50 mM Tris/HCl, pH 7.8) of appropriate deuterium enrichment to provide the desired final isotope composition in the assay. This solution was transferred (1 mL each) to 9 mL Wheaton vials, and the vials were sealed with a polytetrafluoroethylene (PTFE) septa with an aluminum cap. The vials were degassed with 12 cycles of 2 min vacuum, followed by 1 min argon. Dithionite (DT, stock 0.5 M in 0.5 M Tris base pH 10.1, final [DT] in assay = 20 mM), 5 mM DT SEC buffer (50 mM Tris/HCl, 200 mM NaCl, pH 7.8), acetylene (1 mL, 1 atm, 40.8 μmol, 10% of assay atmosphere), and Av1 (0.03 mg per assay) were added by gas-tight Hamilton syringe. The appropriate amount of Av2 [component ratio (mol Av2/mol FeMo-cofactor) = 3–25] was added to start the reaction, and the vials were incubated at 30 °C with shaking. The reaction was quenched after 10–30 min by the addition of citric acid (3 M, 1 mL). For quantification of total ethylene, the headspace (50 μL) was injected into a SRI Instruments GC equipped with an activated alumina column (60/80 mesh) operated at 110 °C and a flame ionization detector.

### Gas Chromatography-Mass Spectroscopy Quantification of Ethylene Isotopologues

For quantification of ethylene isotopologues, the headspace (50 μL) of acetylene reduction assays was injected into an HP 5890 Gas Chromatograph with an HP 5972 Mass Spectrometer equipped with a GASPRO PLOT-Q (Restek) column operated at 80 °C. Overlapping ethylene isotopologue mass spectra are deconvoluted using the fragmentation pattern of a C_2_H_4_ standard (see Supporting Information Figures S2 and S3).

### Fourier Transform Infrared Spectroscopy Quantification of Ethylene Isotopologues

Acetylene reduction assay was performed as described above, except the reaction was scaled up 3X and glass beads were used in the Wheaton vial to minimize headspace volume, thereby increasing the concentration of ethylene in the gas phase. An FTIR gas cell with NaCl windows was degassed (10 cycles of vacuum, followed by backfilling with argon) and placed in the sample chamber of a Bruker Vertex 80 FTIR Spectrometer. The sample chamber was purged for 15 min with N_2_, and then, a background scan was run (128 scans, 1 cm^–1^ resolution). The headspace of the assay reaction vial was transferred to the sample cell by vacuum with a cannula. The sample chamber was closed and allowed to purge for 15 min, and then, an FTIR scan was performed. Peak heights of the stretches in the 840–1000 cm^–1^ region, corresponding to the wagging mode of the H–C–H or H–C–D moieties,^37^ were used to quantify the ethylene isotopologue species. The molar absorptivities of the C_2_H_4_ stretch (949 cm^–1^) and the C_2_H_2_D_2_ stretch (843 cm^–1^) are reported to be equivalent. However, there is a discrepancy in the literature as to whether the C_2_H_3_D stretch (943 cm^–1^) has a molar absorptivity that is the same, or only half, that for C_2_H_4_ and C_2_H_2_D_2_.^23,38,39^ Therefore, a standard curve was performed with C_2_H_4_ and C_2_H_3_D to determine their molar absorptivities as described in Supporting Information Figure S4, which confirmed that the molar absorptivity of the C_2_H_3_D stretch at 943 cm^–1^ is about half of the corresponding peak for C_2_H_4_ and C_2_H_2_D_2_.

### Proton Reduction Assay

The proton reduction assay was performed in the same way as the acetylene reduction assay, except for the omission of acetylene in the reaction vials, and the reaction volume was scaled up. The ATP regeneration system (final concentrations: 15 mM creatine phosphate, 3.8 mM ATP, 3.8 mM MgCl_2_, 50 units/mL creatine phosphokinase) was prepared in the appropriate buffer (50 mM Tris/HCl, pH 7.8) to provide the desired final isotope composition in the assay. This solution was transferred (2.2 mL each) to 9 mL Wheaton vials (equipped with glass beads to take up space), and the vials were sealed with PTFE septa with aluminum caps. The vials were degassed with 12 cycles of 2 min vacuum, followed by 1 min argon. DT (final concentration = 20 mM), 5 mM DT SEC buffer (50 mM Tris/HCl pH 7.8, 200 mM NaCl), and Av1 (0.09 mg Av1 per assay) were added by a gas-tight Hamilton syringe. The appropriate amount of Av2 (component ratio = 15) was added to start the reaction (total reaction volume = 3 mL), and the vials were incubated at 30 °C with shaking. The reaction was quenched after 10–60 min by the addition of citric acid (3 M, 1 mL). For quantification of total L_2_, the headspace (100 μL) was injected into a HP 5890 GC equipped with a molecular sieve 5 Å column operated at 110 °C and a thermal conductivity detector. H_2_ standard curves were performed with each assay.

### ^1^H NMR Quantification of H_2_ and HD

The headspace (3 mL) from the proton reduction assay was transferred using a gas-tight Hamilton syringe to an NMR tube filled with CDCl_3_ (1 mL) and equipped with PTFE septa, bubbling the headspace through the CDCl_3_. ^1^H NMR spectra were taken on a Varian 600 MHz Spectrometer with a 5 mm inverse triple resonance probe with deuterium decoupling. The relaxation delay was 16 s, the pulse angle was 90°, and at least 256 scans were performed. The H_2_ and HD peaks were confirmed with standards. Baseline correction, phase shifting, and peak fitting were performed in MestreNova, and the areas under the fitted peaks were used to determine the relative quantities of H_2_ and HD. The peak area of H_2_ was divided by two to convert the concentration of H nuclei to the concentration of H_2_. Unfortunately, ^2^H NMR was not sensitive enough to detect D_2_ from our assays. H_2_ and HD standards were used to test the isotope fractionation associated with transferring the assay headspace using a Hamilton syringe, and the variation of the H_2_-to-HD ratio with multiple transfers was significantly less than the standard deviation of our measurements at all levels of deuterium enrichment studied.

### NaCN and CH_3_NC Reduction Assays

The sodium cyanide reduction assays were performed in a similar manner to the proton and acetylene reduction assays. The ATP regeneration system (final concentrations: 15 mM creatine phosphate, 3.8 mM ATP, 3.8 mM MgCl_2_, 19 units creatine phosphokinase) was prepared in buffer (50 mM Tris/HCl pH 7.8) of appropriate deuterium enrichment to provide the desired final isotope composition in the assay. This solution was transferred (2 mL each) to 9 mL Wheaton vials, and the vials were sealed with PTFE septa with an aluminum cap. The vials were degassed with 12 cycles of 2 min vacuum, followed by 1 min argon. DT (final concentration = 20 mM), 5 mM DT SEC buffer (50 mM Tris/HCl pH 7.8, 200 mM NaCl), NaCN or CH_3_NC (100 μL, 50 mM in 5 mM DT SEC, 2 μmol, final concentration in assay 2 mM), and Av1 (0.125 mg per assay) were added by a gas-tight Hamilton syringe. Av2 (component ratio = 4) was added to start the reaction, and the vials were incubated at 30 °C with shaking. The reaction was quenched after 30 min by the addition of citric acid (3 M, 1 mL). For quantification of total methane, the headspace (50 μL) was injected into an SRI Instruments GC equipped with an activated alumina column (60/80 mesh) operated at 110 °C and a flame ionization detector.

### Gas Chromatography-Mass Spectroscopy Quantification of Methane Isotopologues

For quantification of methane isotopologues, the headspace (50 μL) from the NaCN and CH_3_NC reduction assays was injected into a HP 5890 Gas Chromatograph with a HP 5972 Mass Spectrometer equipped with a molecular sieve 5 Å column (Restek) column operated at 70 °C with splitless injection. Mass spectra were generated by integrating under the methane peak of the extracted ion chromatograms at *m*/*z* = 14–20. Overlapping methane isotopologue mass spectra were deconvoluted using the fragmentation pattern of a CH_4_ standard in an analogous manner to the method used for ethylene mass spectra (see Supporting Information).

## Results

**Figure 1:**
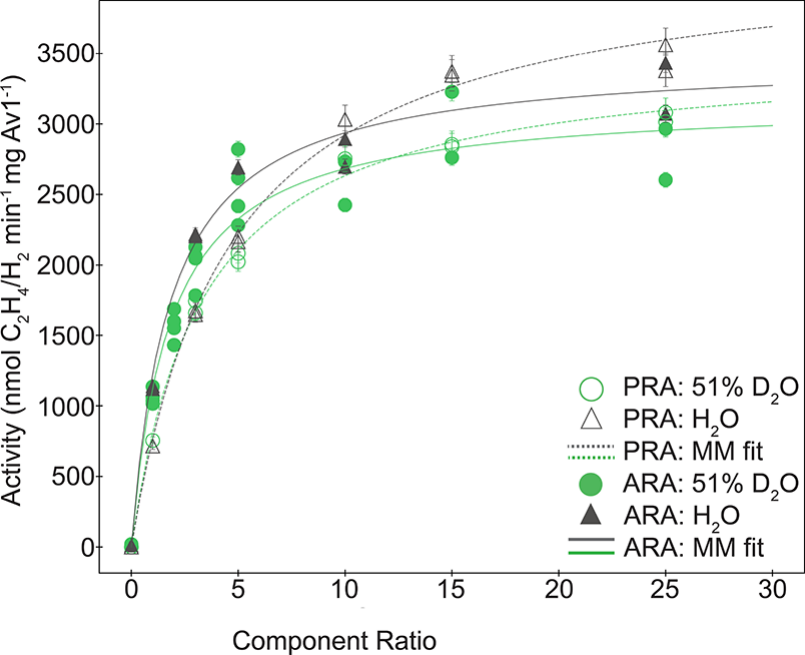
Total acetylene (ARA) and proton (PRA) reduction activity of nitrogenase in mixed isotope buffers. Acetylene (solid markers, solid lines) and proton reduction assays (hollow markers, dashed lines) were performed separately as described in Experimental Section. Standard curves were produced by preparing assay vials in the same method as experimental samples omitting protein and injecting known volumes of ethylene or dihydrogen. Standard curves were used to calculate the activity (nmol C_2_H_4_/H_2_ per mg Av1 per min). The component ratio is defined as the ratio of moles of Fe protein to moles of FeMo-cofactor. Error bars on points are calculated from the relative error of two replicates of standard curves. Data were fit to the Michaelis–Menten equation:  =  nmol min^–1^ mg Av1^–1^;  = ;  =  nmol min^–1^ mg Av1^–1^;  = ;  =  nmol min^–1^ mg Av1^–1^;  = ;  =  nmol min^–1^ mg Av1^–1^;  = , where the units for *K*_m_ are defined in terms of the component ratio.

We first confirmed literature reports^23,33^ demonstrating the absence of a significant solvent kinetic isotope effect on the specific activity of nitrogenase using acetylene, proton, and cyanide as substrates. In both acetylene and proton reduction assays, the maximal rates of ethylene or H_2_ produced in H_2_O versus 51% D_2_O agreed to be within 10–20% (Figure 1), although it is to be noted that the rate in H_2_O was somewhat larger for both substrates. Similarly, in cyanide reduction assays, the rate of methane production was comparable when performed in 100% H_2_O versus 72% D_2_O (Supporting Information Figure S11).

**Figure 2:**
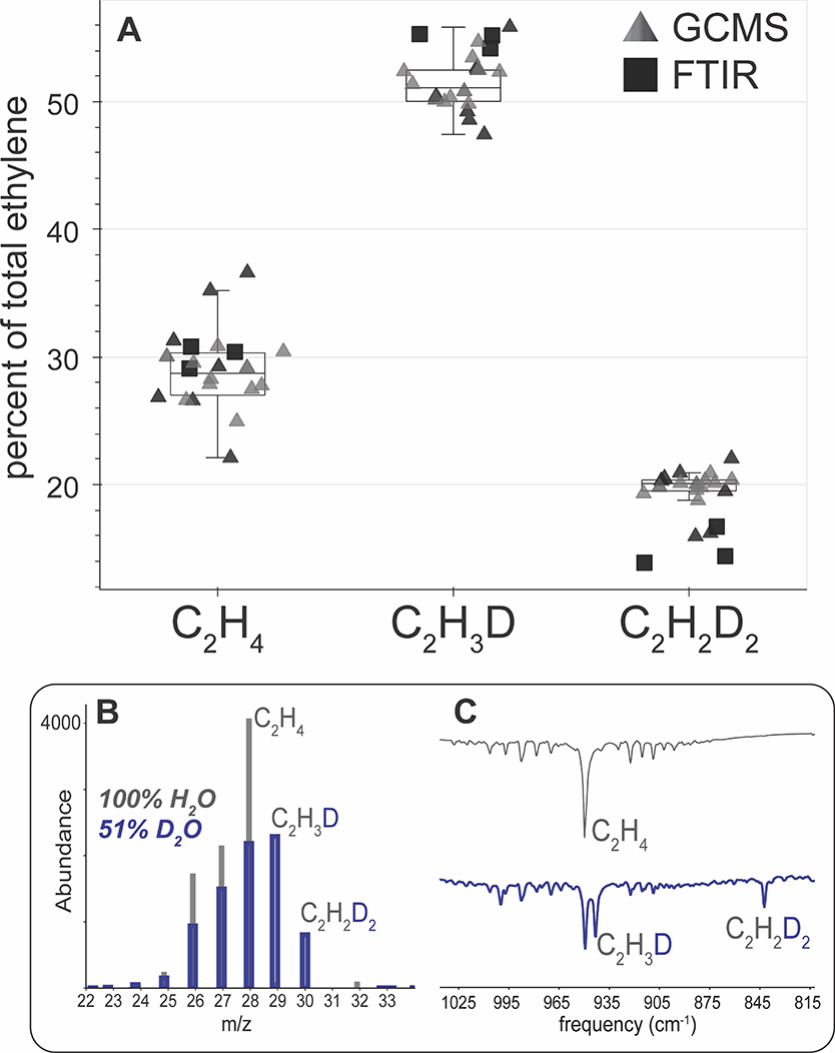
Quantification of isotopologue products of nitrogenase acetylene reduction assays in 51% D_2_O. Top panel (A) shows overlay of results of all assays in 51% D_2_O. Small black and gray triangles are data from GC–MS quantification where each triangle is the result of quantification of one assay vial. The different shades of the triangles represent different component ratios (CRs) used in those assays (20 replicates, CR = 3–25). Black squares represent the data from assays quantified by FTIR (CR = 25, 4 replicates). Bottom panel shows representative GC–MS (B) and FTIR (C) of headspace of acetylene reduction assays in 100% H_2_O (gray) or 51% D_2_O (blue).

Gas chromatography-mass spectroscopy (GC–MS) and Fourier transform infrared spectroscopy (FTIR) were used to detect and quantify the ethylene isotopologue products of acetylene reduction by nitrogenase in 51% D_2_O buffer (Figure 2). In the mass spectra of the ethylene peak from assays in 100% H_2_O (Figure 2B, gray), the molecular ion was at *m*/*z* = 28, as expected for the C_2_H_4_ product, and the magnitude of the abundance observed at *m*/*z* = 29 was 2% of the molecular ion, consistent with the 1.1% natural abundance of ^13^C. The ethylene mass spectra obtained from assays performed in 51% D_2_O (Figure 2B, blue) showed significant abundance at both *m*/*z* = 29 and *m*/*z* = 30 that were not present in the spectra of C_2_H_4_ alone, indicating the formation of C_2_H_3_D and C_2_H_2_D_2_ as products of nitrogenase upon deuterium enrichment of the buffer. Minimal abundance at *m*/*z* = 31 (ranging from undetectable to 5% of the molecular ion peak) was probably due to the natural abundance of ^13^C in the products. As expected, a peak was not observed at *m*/*z* = 32, indicating that C_2_D_4_ was not formed. The overlapping mass spectra of C_2_H_4_, C_2_H_3_D, and C_2_H_2_D_2_ were deconvoluted as described in the Supporting Information, and the distribution of products was determined to be 28 ± 2% C_2_H_4_, 52 ± 2% C_2_H_3_D, and 20 ± 1% C_2_H_2_D_2_ (*cis*- and *trans*-*d*_2_-ethylene could not be distinguished by GC–MS). The *m*/*z* = 27 abundance of the acetylene peak was monitored to determine the extent of H/D exchange in the substrate pool, revealing that C_2_HD made up  1% of the total acetylene on the timescale of our assays.

Figure 2A shows the relative quantities of the ethylene isotopologues produced from acetylene reduction assays in 51% D_2_O. The ratio of C_2_H_4_/C_2_H_3_D/C_2_D_2_D_2_ produced is 1:1.9 ± 0.2:0.73 ± 0.09. If isotope discrimination by nitrogenase resembled a binomial distribution in which the probability of incorporating a hydrogen atom instead of a deuterium atom was 1/2, we would expect a 1:2:1 ratio of products in a 50% D_2_O solution. Therefore, the relative amounts of ethylene isotopologue products that we observe in an approximately 50% D_2_O solution (51% D_2_O) represent a slight depletion of deuterium into the products relative to the solvent. These data are the average of 20 trials performed at varying component ratios (Figure 2A, black and gray triangles). No significant difference in distribution of isotopologue products was observed upon variation of the component ratio, indicating that the rate of electron flow to the active site of the MoFe protein does not affect the isotope discrimination during reduction.

FTIR spectroscopy was also used to quantify the results of acetylene reduction assays to confirm the quantification determined by GC–MS (Figure 2A, black squares; Figure 2C). Stretching frequencies at 943 and 843 cm^–1^ were observed by FTIR in acetylene reduction assays performed with deuterium enrichment of the buffer but not in assays without D_2_O, corresponding to the presence of C_2_H_3_D and *cis*-C_2_H_2_D_2_, respectively. The peak heights of the FTIR stretches were used to quantify the products as described in Experimental Section, and the distribution of ethylene isotopologues was 31 ± 1% C_2_H_4_, 54 ± 1% C_2_H_3_D, and 14 ± 2% C_2_H_2_D_2_, agreeing well with the results obtained by GC–MS. The FTIR stretch from *trans*-C_2_H_2_D_2_ at 988 cm^–1^ could not be observed, indicating that the levels of the *trans*-C_2_H_2_D_2_ product were very low. Previous reports^23^ have shown that, in 100% D_2_O, nitrogenase makes about 96% *cis*-C_2_H_2_D_2_ and only about 4% *trans*-C_2_H_2_D_2_ which is consistent with the undetectable levels of *trans*-C_2_H_2_D_2_ in the headspace of our assays.

**Figure 3:**
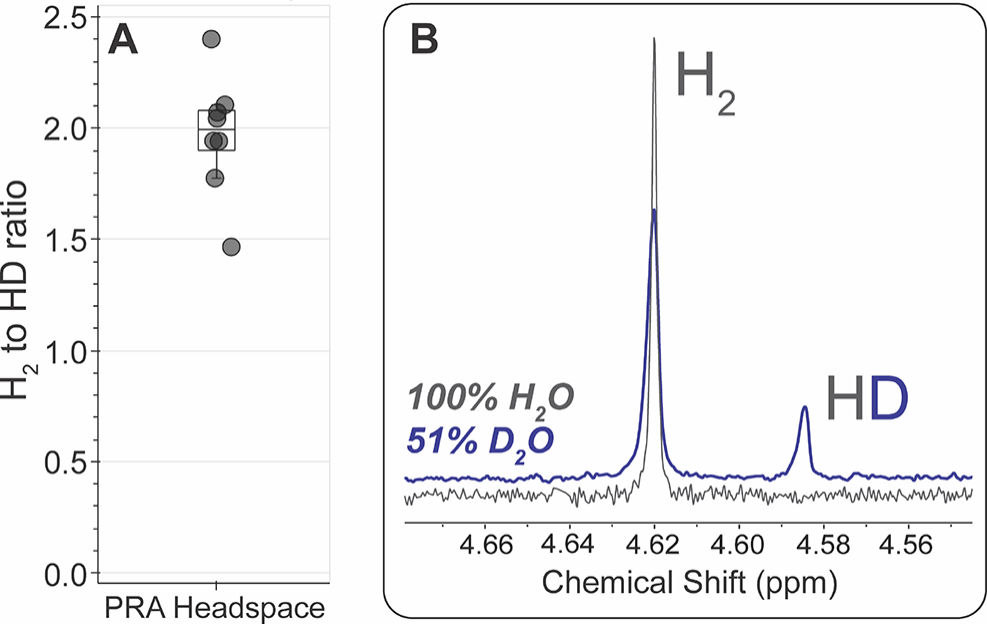
Quantification of isotopologue products of nitrogenase proton reduction assays in 51% D_2_O. Left panel (A) shows overlay of results of all assays in 51% D_2_O. Each circle represents the H_2_-to-HD ratio of one proton reduction assay trial. Average H_2_-to-HD ratio produced in all trials at 51% D_2_O is 2.0 ± 0.3. Right panel shows representative ^1^H NMR spectra of headspace of proton reduction assays in 100% H_2_O (gray) or 51% D_2_O (blue).

In contrast to the results of acetylene reduction assays, proton reduction assays showed significant deuterium fractionation during H_2_ production (Figure 3A). Analogously to the acetylene reduction experiments, proton reduction assays were performed in either H_2_O or 51% D_2_O, and the products in the headspace were quantified by ^1^H NMR. A 1:1:1 triplet corresponding to HD was observed at δ 4.82 ppm from assays in 51% D_2_O that was absent in assays performed in 100% H_2_O (Supporting Information Figure S6). Deuterium decoupling was employed to compress the triplet into a singlet for more accurate quantification of the HD peak (Figure 3B). The ratio of H_2_ to HD in the headspace of proton reduction assays of nitrogenase was quantified by NMR to be 2.0 ± 0.3 (8 replicates), indicating a significant depletion of deuterium in the products relative to the solvent. H_2_ production during the acetylene reduction assays was not observed.

**Figure 4:**
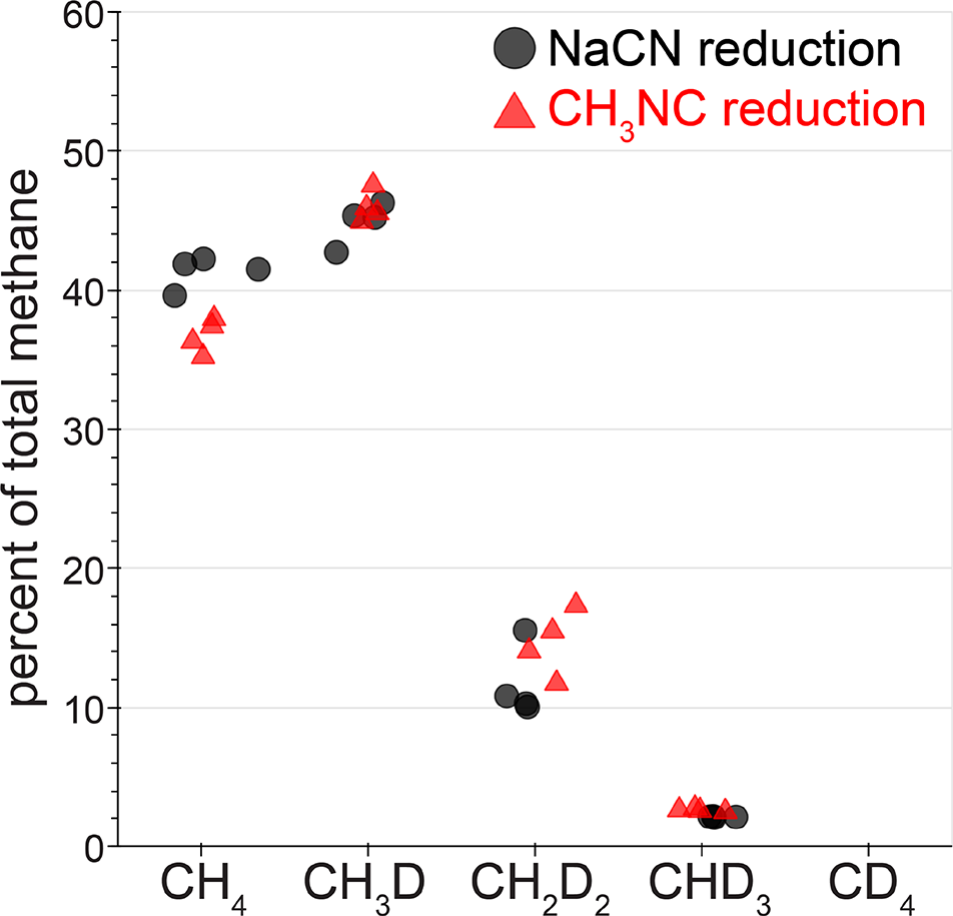
Quantification of methane isotopologue products from the nitrogenase-catalyzed reductions NaCN and CH_3_NC in 51% D_2_O. Each black circle or red triangle are the result of the quantification of the indicated methane isotopologue (*x*-axis) of a single NaCN or CH_3_NC reduction assay trial, respectively. No CD_4_ was observed.

Similar to the results of proton reduction assays, significant deuterium depletion was observed in the methane products of sodium cyanide and methyl isocyanide reduction by nitrogenase. The distribution of methane isotopologues was quantified in the headspace of these assays by GC–MS in a manner analogous to that of the quantification of ethylene isotopologues from acetylene reduction assays (Supporting Information Figure S12). In 100% H_2_O, the molecular ion of the methane peak was observed at *m*/*z* = 16, consistent with the molecular weight of CH_4_, with abundance at *m*/*z* = 15 and *m*/*z* = 14 from fragments formed during ionization. When the NaCN and CH_3_NC reduction assays were performed in 51% D_2_O, abundance was observed in the methane mass spectra at *m*/*z* = 17, *m*/*z* = 18, and *m*/*z* = 19, indicating that CH_3_D, CH_2_D_2_, and CHD_3_ were formed, respectively. The distribution of methane products from NaCN reduction assays in 51% D_2_O was 41 ± 1% CH_4_, 45 ± 2% CH_3_D, 12 ± 3% CH_2_D_2_, and 2.13 ± 0.04% CHD_3_. The distribution of methane isotopologues produced from methyl isocyanide reduction assays (37 ± 1% CH_4_, 46 ± 1% CH_3_D, 15 ± 2% CH_2_D_2_, 2.6 ± 0.1% CHD_3_) was similar to that observed from cyanide reduction assays (Figure 4, Table S4).

Next, a proton inventory analysis was performed, in which the deuterium enrichment was varied (25, 51, and 73–75% D_2_O) and the relative amounts of isotopologue products were quantified (distribution of products from all assays reported in Supporting Information; acetylene reduction: Tables S1 and S2; proton reduction: Table S3; cyanide reduction: Table S4). From the relative distribution of isotopologue products of acetylene, proton, and cyanide reduction assays, the isotope effects of hydrogen/deuterium addition were determined for these processes. Under conditions when a process involves two proton/deuteron transfer steps, each with the same isotope effect, the mole fraction of each of the isotopologue products can be expressed in terms of the isotope effect and the deuterium enrichment of the solvent by the following equations (see Supporting Information for derivation)^40,41^
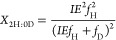
1
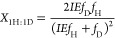
2
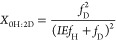
3where *X*_2H:0D_, *X*_1H:1D_, and *X*_0H:2D_ are the mole fractions of the isotopologue product with 2 Hs (C_2_H_4_ or H_2_), 1 H and 1D (C_2_H_3_D or HD), or 2Ds (C_2_H_2_D_2_ or D_2_) incorporated, respectively. IE is the isotope effect of H/D addition (*k*_H_/*k*_D_), and *f*_H_ and *f*_D_ are the mole fractions of hydrogen and deuterium in the solvent, respectively. Plots of eqs 1–3 are included in the Supporting Information with the experimental data, revealing the isotope effect value at which the data intercept with the model (Figure S7). Least squares fits of the products of the acetylene reduction assays to eqs 1–3 result in three isotope effect values for each of the ethylene isotopologues (Supporting Information Figure S8), and these values are 1.28 ± 0.04, 1.4 ± 0.1, and 1.59 ± 0.05 for C_2_H_4_, C_2_H_3_D, and C_2_H_2_D_2_, respectively.

To obtain an overall isotope effect value for acetylene reduction by nitrogenase, we can calculate the total amounts of hydrogen versus deuterium atoms incorporated into the ethylene products by the following equations
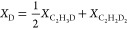
4
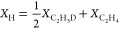
5where *X*_D_ and *X*_H_ are the mole fractions of H versus D incorporated into the reduced products. The mole fraction of deuterium incorporated (*X*_D_) is related to the isotope effect by the following equation
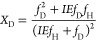
6

**Figure 5:**
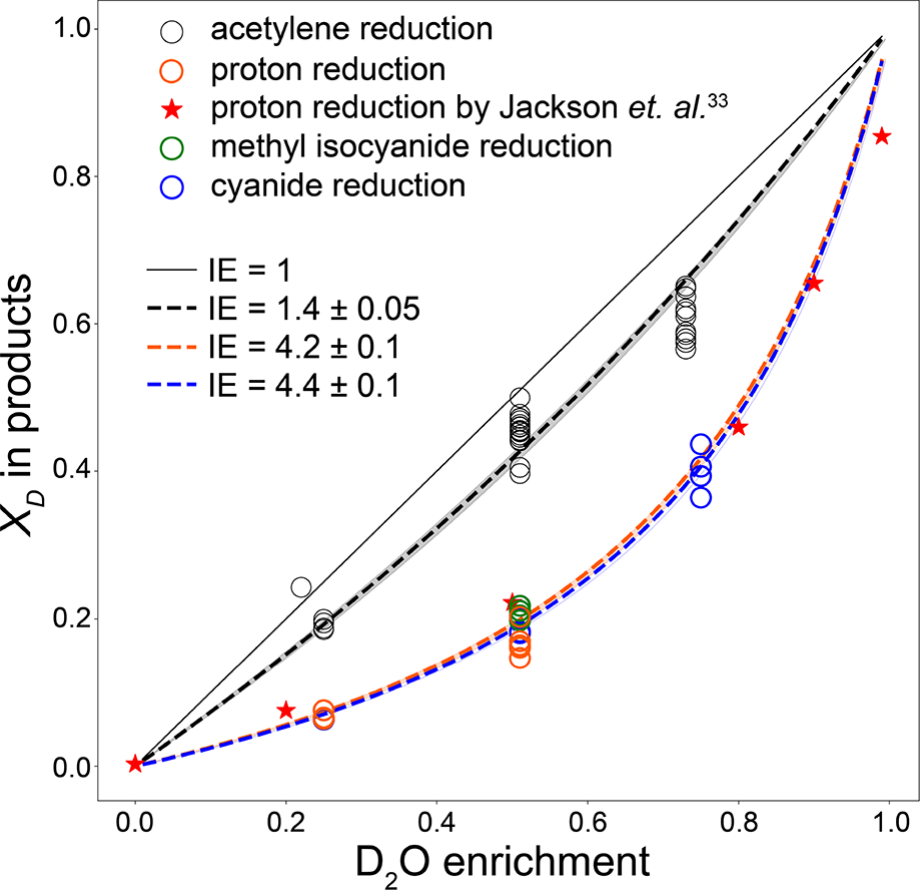
Mole fraction of deuterium incorporated into products during acetylene (black circles), proton (blue circles), and cyanide reduction (orange circles) as a function of deuterium enrichment of the solvent. Least squares fit of experimental data to eq 6 (dashed lines, uncertainty of least squares fit shown as shading) produced values for the isotope effect during acetylene (black) and proton reduction (orange). For the proton reduction values, only data from 25% D_2_O and 51% D_2_O are shown, assuming negligible D_2_ is produced. Experimental data from Jackson et al.^33^ shown as red stars and least squares fit of these data are indistinguishable from the fit to our proton reduction data. For cyanide reduction, a least squares fit of the experimental data against eq 12 produced a value of 4.4 ± 0.1 for the isotope effect (blue dashed line). Results of reduction of methyl isocyanide at 51% D_2_O shown as green circles.

A least squares fit of eq 6 to the experimental data from the acetylene reduction assays resulted in an isotope effect of 1.40 ± 0.05 (Figure 5, Table 1), which agrees with the values obtained from the individual least squares fits of eqs 1–3 (Figure 5, Table 1, Supporting Information Figure S8).

**Table 1:** Isotope Effects of Acetylene, Cyanide, and Proton Reduction by Nitrogenase

	acetylene reduction	proton reduction	cyanide reduction
isotope effect	1.40 ± 0.05	4.2 ± 0.1	4.4 ± 0.1

For cyanide and methyl isocyanide reduction, 4 H atoms are incorporated into the methane product, introducing further opportunities for deuterium fractionation. The mole fraction of each methane isotopologue product can be expressed as a function of the isotope effect by the following formulas
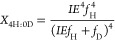
7
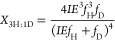
8
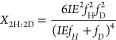
9
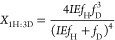
10
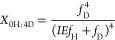
11where *X*_4H:0D_, *X*_3H:1D_, *X*_2H:2D_, *X*_1H:3D_, and *X*_0H:4D_ are the mole fraction of CH_4_, CH_3_D, CH_2_D_2_, CHD_3_, and CD_4_, respectively, produced in cyanide and methyl isocyanide reduction assays. Plots of these equations are included in the Supporting Information (Figure S7) with the experimental values for mole fraction of methane isotopologue produced shown at the isotope effect where they intercept the models. Least squares fit of each of these models to the experimental data was performed, and the isotope effects obtained are shown in Supporting Information Figure S10.

Analogously to the analysis of the acetylene reduction data, a single expression can be written for the mole fraction of deuterium added during methane production to obtain an overall value for the isotope effect of this process
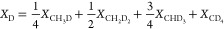
12where *X*_D_ is the mole fractions of D incorporated into the reduced products. To obtain an expression for the mole fraction of deuterium incorporated (*X*_D_) as a function of the isotope effect, eqs 7–11 are inserted into eq 12. A least squares fit of eq 12 to the experimental data from the cyanide reduction assays resulted in an isotope effect of 4.4 ± 0.1 (Figure 5, Table 1), which agrees with the values obtained from the individual least squares fits of eqs 7–11 (Figure S10) and is significantly greater than the isotope effect observed during acetylene reduction.

For the proton reduction assays, we were unable to quantify the relative amounts of D_2_ by either GC–MS or NMR, so it is not possible to use eqs 1–3 to obtain isotope effects for the proton reduction data. However, the ratio of H_2_ to HD can be calculated from the ^1^H NMR data, and this ratio can be written in terms of the isotope effect and the deuterium enrichment of the solvent
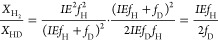
13

Equation 13 is plotted in Supporting Information Figure S7 (black line, right axis), and the experimental data for the H_2_/HD produced in proton reduction assays are shown (black circles) at the isotope effect where they intercept with the model. Like those observed during cyanide reduction, the isotope effects observed from proton reduction assays were much greater than those seen during acetylene reduction. The H_2_-to-HD ratio observed from proton reduction assays was 6.3 ± 0.6 in 25% D_2_O, 2.0 ± 0.3 in 51% D_2_O, and 0.9 ± 0.1 in 73% D_2_O, which results in isotope effects of 4.2 ± 0.4, 4.1 ± 0.5, and 4.9 ± 0.5, respectively. A least squares fit was performed on the experimental H_2_/HD ratio of products observed against the deuterium enrichment of the solvent with eq 13, and the isotope effect obtained from this fit was 4.2 ± 0.1 (Table 1, Supporting Information Figure S9).

In modeling these data, we have assumed that the isotope effect describing the relative rates of incorporation of H versus D is a constant for a given substrate, independent of how many H/D have been previously incorporated. Models were also constructed with two distinct isotope effects for each of the two H/D atoms incorporated in ethylene/H_2_; however, the least squares fits of the data to these models were very similar to the aforementioned models with a single isotope effect for both H/Ds added, so only the latter values are reported. We also note that the isotope effects calculated for the individual ethylene isotopologues generated during the reduction of acetylene (Figure S8) increase with increasing D content and that the fraction of C_2_H_2_ and C_2_H_2_D_2_ present in 72% D_2_O is over- and under-estimated based on the simple model. These observations are perhaps suggestive that the H/D transfers are not completely independent and/or that the detailed reaction kinetics are sensitive to the level of deuteration, which could also impact protein structure dynamics at higher levels. In view of reasonable fit to all the experimental data using a single isotope effect for each substrate (Figure 5; Table 1), we conclude that the simple model assuming each H/D transfer is independent of previous transfers and that the isotope effect is independent of the solvent isotopic composition represents a reasonable level of approximation.

There is no significant exchange between the protons of ethylene, dihydrogen, or methane with water in the time scale of the assays (tested by analyzing the headspace at various time points up to 1 h after quenching), so the isotopologue products in the headspace are an accurate reflection of the products of nitrogenase.

## Discussion

In agreement with previous reports, our assays exhibited no significant solvent isotope effect on the specific activity of total substrate reduction by nitrogenase. This result is consistent with the rate-determining step for nitrogenase being something other than substrate reduction, namely, release of inorganic phosphate from ATP during the Fe protein cycle.^28^ This masking of any potential isotope effects on substrate reduction by slower processes has precluded the use of kinetic isotope experiments to probe the mechanism of nitrogenase. However, because the reduction performed by nitrogenase is irreversible, an isotope effect can be determined from the extent of incorporation of nonexchangeable deuterium into the products upon deuterium enrichment of the solvent. In this work, we measured the incorporation of H/D from solvent into products to determine the isotope effect associated with acetylene, proton, and cyanide reduction by Mo-nitrogenase from *A. vinelandii*. Various analytical chemistry techniques were used to quantify the isotopologue products of these reactions. There is a slight depletion of deuterium in the ethylene products of acetylene reduction, corresponding to an isotope effect of 1.40 ± 0.05, whereas significant deuterium depletion is observed in the H_2_ and CH_4_ products of the proton and cyanide reduction assays, with isotope effects of 4.2 ± 0.1 and 4.4 ± 0.1, respectively.

In calculating isotope effects, we have used a model where the probability of H or D incorporation is independent of how many H/D transfers have already occurred. The reactions studied in this work are reasonably well fit by a binomial distribution with isotope effects relatively insensitive to solvent composition (Figure 5). Application of this binomial model to the two H/D transfers associated with acetylene and proton reduction, under conditions where the only products were ethylene and H_2_, respectively, was straightforward. There were several complexities in treating methane formation from cyanide and methyl isocyanide, however. Methane represents only one of several products that can form during the reduction of these substrates; for example, cyanide reduction produces ammonia (with methane formation) and methylamine, while methyl isocyanide can also produce methylamine (with methane formation), dimethylamine, and various C2 and C3 hydrocarbons.^13,16,25^ Although methane formation for both substrates is the result of an overall six electron process (with the carbon changing formal oxidation state from +2 to −4), there are four C–H bonds, so we analyzed the methane isotopologue distribution assuming 4 H/D transfers. For cyanide reduction, there is the added twist that the actual substrate is likely to be the acid, HCN or DCN, rather than the cyanide ion. Relatively small differences in p*K*_a_’s have been reported for HCN and DCN.^42,43^ We explored various models based on binding of the acid form, followed by 3 H/D transfers. Since these fits were no better than those derived for 4 H/D transfers at the expense of an additional parameter (the binding probability of HCN relative to DCN), we utilized the 4 H/D transfer approach to obtain the associated isotope effect.

Our observations of the isotopic effects associated with dihydrogen production by nitrogenase are in general agreement with previous reports. A pioneering study by Hardy and co-workers quantified H_2_, HD, and D_2_ produced by partially purified nitrogenase in varying mole fractions of D_2_O in the solvent, and our results are similar to what they observed.^33^ Least squares fits of our data and the Hardy results with eq 6 are indistinguishable within error (Figure 5). A more recent report took a different approach to determining the kinetic isotope effect of H_2_ production by eliminating the need for the slow steps of the Fe protein that mask the kinetic isotope effects at the active site. In this work, the MoFe protein was immobilized on an electrode surface and directly reduced with the aid of a redox mediator.^44^ By measuring the catalytic current of H_2_ production in varying levels of D_2_O enrichment in the buffer, a kinetic isotope effect of about 2.7 was observed. An isotope effect for H_2_ evolution by nitrogenase was also observed spectroscopically by irradiation of the FeMo-cofactor (with 4 H atoms bound) at 450 nm. This photoinduced reductive elimination of H_2_ and subsequent oxidative addition of H_2_ upon annealing showed large kinetic isotope effects of about 10 and 5, respectively; interpreted as evidence that both of these processes involve an energy barrier and that hydrogen tunneling could be involved.^45,46^

Similar to our cyanide reduction results, a large hydrogen isotope effect was also observed^47^ for methane production from CO_2_ by the vanadium- and iron-only nitrogenases in *Rhodopseudomonas palustris*. The hydrogen isotope fractionation was measured to be 2.1, the largest reported for any biogenic and geogenic methane evolution pathway. Interestingly, the ^13^C fractionation observed from methane production by this organism was more modest and within the range of other microbial methanogenesis. Beyond the different substrates (NaCN instead of CO_2_), other significant differences between our studies and this report include the latter study measured *in vivo* fractionation in cultures of *R. palustris* expressing different nitrogenases, rather than using purified Mo-nitrogenase from *A. vinelandii*. Nevertheless, these reports suggest that deuterium depletion could be a common feature of methane production by nitrogenase.

Our finding that the solvent isotope effects can vary significantly for the reduction of different nitrogenase substrates indicates that the details of the hydrogen transfer can also vary. There is precedent for this in the observation that pH-rate profiles for acetylene and proton reduction are shifted relative to each other.^48^ The H atoms in nitrogenase products could be plausibly derived from H_2_O, H_3_O^+^ (or an acidic protein group), a protonated cluster sulfur, or hydrides on the FeMo-cofactor. If H atoms are sourced directly from H_2_O, without an isotope effect on binding or transfer, the deuterium enrichment of the product would reflect the deuterium enrichment of the solvent and would thus result in an isotope effect close to unity. Protons from H_3_O^+^ will result in products depleted in deuterium with an isotope effect consistent with the difference in p*K*_a_ between H_2_O and D_2_O,^49^ which is the explanation provided by Hardy and co-workers for the aforementioned isotope effect that they observed during proton reduction by partially purified nitrogenase.^33^ More generally, the substitution of D for H typically shifts the p*K* values by 0.5 units, and so, a similar isotope effect might be expected for proton donation from an amino acid side chain as from H_3_O^+^.^49^

Sourcing H atoms from hydrides on the FeMo-cofactor will likely also exhibit an isotope effect. In a synthetic FeFe complex with a terminal thiolate ligand and a bimetallic center, H/D exchange between the bridging hydride and the proton on the sulfur was studied.^50^ This report found that H/D exchange between the protonated sulfur and the solvent was fast because the thiol is acidic, while the H/D exchange on the hydride was slow. The *K*_eq_ of the interconversion of the complex with deuterium at the hydride position and hydrogen on the sulfur to the complex with hydrogen at the metal hydride position and deuterium on the sulfur was measured to be 2.36, indicating that a 50% D_2_O solution will produce complexes in which 70% of the bridging metal hydrides are occupied by hydrogen and 30% are occupied by deuterium.

From kinetic studies looking at product formation as a function of electron flux through the system, cyanide, methyl isocyanide, and acetylene have been found to bind redox states of the MoFe protein more oxidized than those implicated in H_2_ evolution and N_2_ reduction.^14,15^ In the Thorneley–Lowe kinetic model for nitrogenase, the MoFe protein cycles through a series of states, designated E_*n*_, where *n* is the number of electrons transferred from the Fe protein, relative to the as-isolated, resting state of the MoFe protein. Cyanide, isocyanide, and acetylene can bind to the E_1_ state (and potentially E_0_), while H_2_ evolution occurs at states E_2_, E_3_, and E_4_, and N_2_ binding occurs at E_3_ and E_4_.^9,13^ Despite the similarities in the E_*n*_ state binding of cyanide, methyl isocyanide, and acetylene, our results show that their reductions result in significantly different levels of deuterium incorporation. Furthermore, while hydrogen evolution takes place from a more highly reduced state, the isotope effect of hydrogen evolution is similar to that of cyanide and methyl isocyanide reduction and quite different from that of acetylene reduction. Therefore, the reduction state of nitrogenase at which a particular substrate binds is not the sole determining factor of the H/D isotope effect during substrate reduction.

Here, we report the striking result that the H/D isotope discrimination of acetylene reduction is significantly different from that of proton and cyanide reduction. More generally, these results reveal differences in the detailed H atom transfer reactions accompanying the reduction of different substrates by nitrogenase. This work not only deepens our understanding of the complex reactions at the active site but also provides a new strategy for analyzing the nitrogenase mechanism with product deuterium isotope effect experiments.

## Supporting Information Available

Additional spectra of isotopologue products, derivation of isotope effect model equations, least squares fits of experimental data to the models, and description of processing mass spectra (PDF)
