# Supplementary material for: Solvent Deuterium Isotope Effects of Substrate Reduction by Nitrogenase from Azotobacter vinelandii
Source: J Am Chem Soc. 2022 Nov 8;144(46):21125–35. doi: 10.1021/jacs.2c07574 (PMC9706557; doi:10.1021/jacs.2c07574)
Supplement: Supplementary file 1 — ja2c07574_si_001.pdf [file ja2c07574_si_001.pdf]

Supplemental Information: Solvent Deuterium Isotope Effects of Substrate Reduction by Nitrogenase from Azotobacter vinelandii

Siobhán G. MacArdle and Douglas C. Rees*

California Institute of Technology, Howard Hughes Medical Institute, Department of Chemistry and Chemical Engineering, Pasadena, California, 91125, USA

E-mail: dcrees@caltech.edu

Nitrogenase Purification

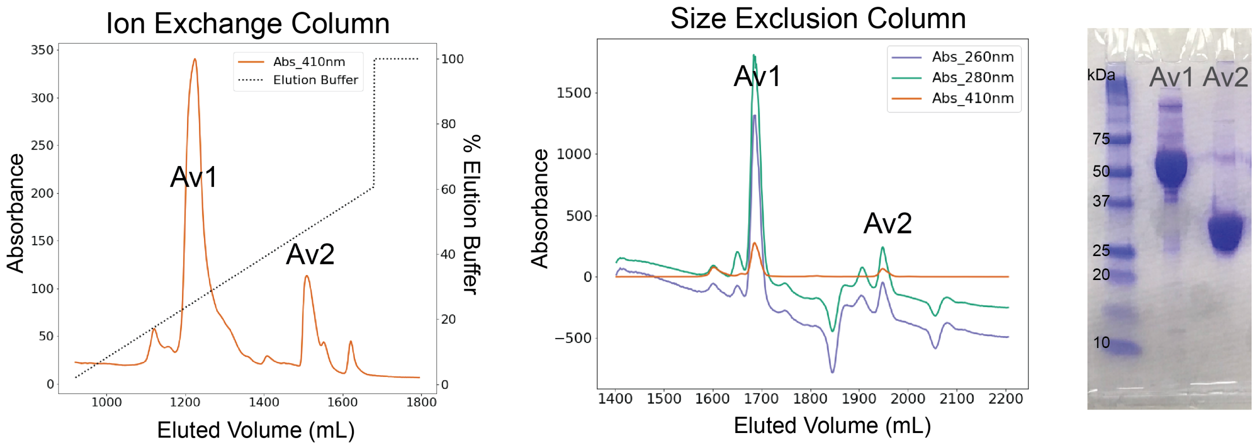

Figure S1: Representative chromatography traces and denaturing gels from purification of nitrogenase enzyme from *Azotobacter vinelandii*. Av1 and Av2 purified using ion exchange chromatography (left) followed by size exclusion chromatography (middle). Details of purification described in *Methods*. Av1 collected from ion exchange column at 19-22.5% Elution Buffer (312-341 mM NaCl) and Av2 collected from ion exchange column at 37.5-40.8% Elution Buffer (469-497 mM NaCl). Av1 collected from size exclusion at 1671-1703 mL and Av2 collected from size exclusion column at 1940-1971 mL. To run gel (BioRad Mini-PROTEAN TGX Stain Free Gels), 15 µL sample was combined with 5 µL of loading dye and 11 µL of the mixture was loaded into each well. Gel ran at 200 V for 20 minutes then rinsed with milliQ water, stained with Coomassie blue, and destained with a 40% methanol 10% acetic acid solution.

Gas Chromatographs of Headspace of Acetylene Reduction Assays

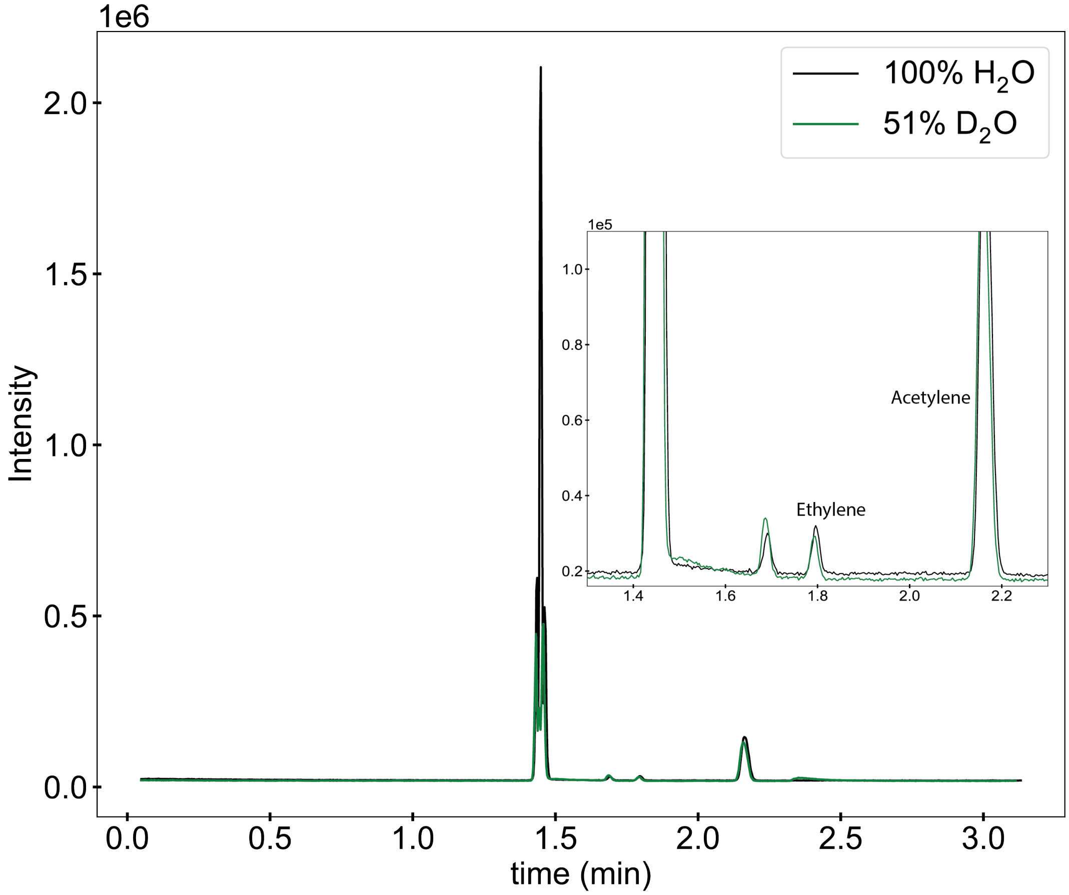

Figure S2: Representative gas chromatogram of headspace of acetylene reduction assay of nitrogenase in 100% H_2 (black) or 51% D_2O (green). Inset is zoom of region where ethylene peak elutes (1.8 min). Assay and GC-MS quantification of ethylene isotopologues described in *Methods*. Headspace (50 µL) injected using a Hamilton gastight syringe. Column : GASPRO PLOT-Q (Restek), column temperature: 80 °C (isothermal).

Mass Spectra of Ethylene Peak of Headpsace of Acetylene Reduction Assays

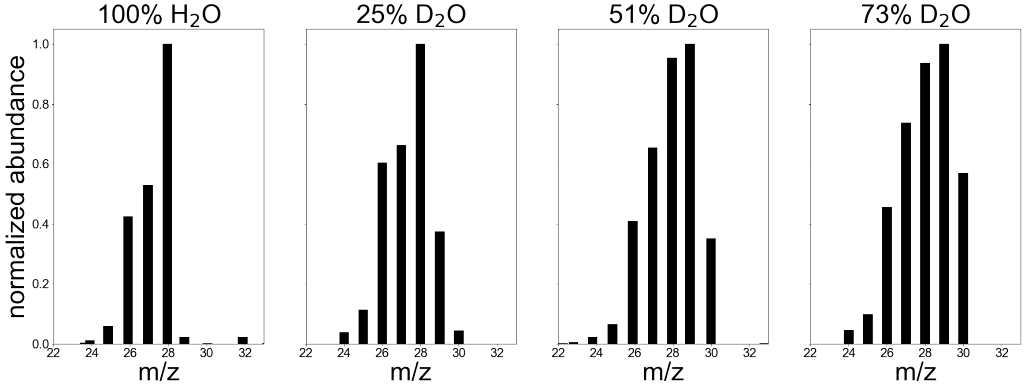

Figure S3: Mass spectra of ethylene peaks in the headspace of acetylene reduction assays in 100% H_2O, 25% D_2O, 51% D_2O, and 73% D_2O. Abundance normalized to peak of greatest abundance. Mass spectra generated by integrating under ethylene peak of extracted ion chromatograms of m/z = 23-32. As the mole fraction of D_2O in buffer increases, greater abundance seen at m/z = 29 and m/z = 30, corresponding to increase in C_2H_3D and $C_2H_2D_2$ in the headspace.

Deconvolution of Overlapping Mass Spectra of Ethylene Isotopologues

Mass spectra of the headspace of acetylene reduction assays in deuterated solvent show abundance at m/z = 28 from C_2H_4, m/z = 29 from C_2H_3D, and m/z = 30 from $C_2H_2D_2$. Quantifying the relative amounts of these species is not as straightforward as simply comparing the abundance at m/z = 28 vs. m/z = 29 vs. m/z = 30 because of the fragmentation that ethylene undergoes upon mass spectrometry analysis at 70 eV. The abundance in the mass spectrum at m/z = 29 is due to the molecular ion of C_2H_3D and also the fragmentation of $C_2H_2D_2$ in which it has lost one atomic mass unit (amu). Similarly, the abundance at m/z = 28 is due to the molecular ion of C_2H_4, the fragments of C_2H_3D that have lost one amu, and the fragments of $C_2H_2D_2$ that have lost two amu. Therefore, these overlapping mass spectra need to be deconvoluted in order to quantify relative amounts of C_2H_4, C_2H_3D,and $C_2H_2D_2$. The method used for this deconvolution is described here.

With each assay, a non-deuterated ethylene standard was used to measure the fragmentation of C_2H_4 and the other ethylene isotopologues were assumed to have the same fragmentation pattern, as has been assumed in previous reports.[1] All abundance values were obtained by integrating under the extracted ion chromatogram of each ion.

First, the abundance at each m/z value for the mass spectra of C_2H_4 was normalized to the molecular ion at m/z = 28 to generate the fraction of the abundance of the molecular ion at that particular m/z value. For example $F(27, C_2H_4)$ is equal to the abundance at m/z = 27 divided by the abundance at m/z = 28 of the same spectra:

$$F(28, C_2H_4) = \frac{A_{C_2H_4}(28)}{A_{C_2H_4}(28)} = 1$$

$$F(27, C_2H_4) = \frac{A_{C_2H_4}(27)}{A_{C_2H_4}(28)}$$

$$F(26, C_2H_4) = \frac{A_{C_2H_4}(26)}{A_{C_2H_4}(28)}$$

$$F(25, C_2H_4) = \frac{A_{C_2H_4}(25)}{A_{C_2H_4}(28)}$$

where $A_{C_2H_4}(m/z)$ is the observed abundance at that m/z value from the ethylene standard analyzed using the same method as the assay samples. For the deuterated ethylene isotopologues, $F(27, C_2H_4)$ is the probability that a molecule of ethylene will fragment with the loss of one H/D relative to the molecular ion. $F(26, C_2H_4)$ is the probability that the ethylene molecule will fragment by the loss of two H/Ds and $F(25, C_2H_4)$ is the probabililty that ethylene will fragment by losing 3 H/Ds.

Next, the abundance values from the mass spectra of the headspace samples of the acetylene reduction assays were deconvoluted by calculating the contribution at each m/z from each isotopologue (written as C(m/z, $C_2H_2L_2$)). To do this, each isotopologue was considered independently. For the dideuterated isotopologue, the molecular ion is m/z = 30 and the only contribution to the abundance at m/z = 30 is the dideuterated ethylene. The fragments of $C_2H_2D_2$ in which one H atom is lost will contribute to the abundance at m/z = 29. Because $C_2H_2D_2$ has 2 H atoms and 2 D atoms, the probability that it will lose one H atom (when it fragments by the loss of one H or D) is 1/2. Therefore, the contribution from $C_2H_2D_2$ at m/z = 29 ($C(29, C_2H_2D_2)$) is 1/2 multiplied by the probability of losing one H/D ($F(27, C_2H_4)$), thus $C(29, C_2H_2D_2) = A(30) \times \frac{1}{2}F(27, C_2H_4)$. This analysis was repeated for each m/z from the molecular ion (m/z = 30) to m/z = 25. Then this analysis was repeated for the singly-deuterated ethylene.

Contribution from $C_2H_2D_2$:

$$C(30, C_2H_2D_2) = A(30)$$

$$C(29, C_2H_2D_2) = A(30) \times \frac{1}{2}F(27, C_2H_4)$$

$$C(28, C_2H_2D_2) = A(30) \times \frac{1}{2}F(27, C_2H_4) + A(30) \times \frac{1}{6}F(26, C_2H_4)$$

$$C(27, C_2H_2D_2) = A(30) \times \frac{4}{6} F(26, C_2H_4)$$

$$C(26, C_2H_2D_2) = A(30) \times \frac{1}{6} F(26, C_2H_4) + A(30) \times \frac{1}{2} F(25, C_2H_4)$$

$$C(25, C_2H_2D_2) = A(30) \times \frac{1}{2} F(25, C_2H_4)$$

Contribution from C_2H_3D:

$$C(29, C_2H_3D) = A(29) - C(29, C_2H_2D_2)$$

$$C(28, C_2H_3D) = C(29, C_2H_3D) \times \frac{3}{4} F(27, C_2H_4)$$

$$C(27, C_2H_3D) = C(29, C_2H_3D) \times \frac{1}{4} F(27, C_2H_4) + C(29, C_2H_3D) \times \frac{1}{2} F(26, C_2H_4)$$

$$C(26, C_2H_3D) = C(29, C_2H_3D) \times \frac{1}{2} F(26, C_2H_4) + C(29, C_2H_3D) \times \frac{1}{4} F(25, C_2H_4)$$

$$C(25, C_2H_3D) = C(29, C_2H_3D) \times \frac{3}{4} F(25, C_2H_4)$$

Contribution from C_2H_4:

$$C(28, C_2H_4) = A(28) - C(28, C_2H_2D_2) - C(28, C_2H_3D)$$

$$C(27, C_2H_4) = A(27) - C(27, C_2H_2D_2) - C(27, C_2H_3D)$$

$$C(26, C_2H_4) = A(26) - C(26, C_2H_2D_2) - C(26, C_2H_3D)$$

$$C(25, C_2H_4) = A(25) - C(25, C_2H_2D_2) - C(25, C_2H_3D)$$

The relative ammounts of C_2H_4, C_2H_3D, and $C_2H_2D_2$ were determined by comparing the

molecular ion peak of the spectra of each isotopologue after subtracting the contribution from the fragments of the other isotopologues ($C(28, C_2H_4)$, $C(29, C_2H_3D)$, and $C(30, C_2H_2D_2)$, respectively). The results are summarized in Table S1.

Table S1: Relative Amounts of Ethylene Isotopologue Products of Acetylene Reduction Assays Quantified by GC-MS

Deuterium Enrichment	C_2H_4	C_2H_3D	$C_2H_2D_2$
25%	65 ± 1%	30.6 ± 0.9%	3.9 ± 0.2%
51%	28 ± 2%	52 ± 2%	20 ± 1%
73%	13 ± 3%	49 ± 2%	37 ± 2%

FTIR Standard Curves of Deuterated Ethylenes

The FTIR spectra of the headspace of acetylene reduction assays contained sharp stretches in the 840 -1000 cm^{-1} region, corresponding to the wagging mode of the H-C-H or H-C-D moieties.[2] The peak heights of these stretches were used to quantify the relative amounts of ethylene isotopologue products from acetylene reduction assays. It is well reported that the molar absorptivity of the C_2H_4 stretch (949 cm^{-1}) and the $C_2H_2D_2$ stretch (843 cm^{-1}) are equivalent. However, there is some discrepancy as to whether the C_2H_3D stretch (943 cm^{-1}) has a molar absorptivity that is half of that of C_2H_4 and $C_2H_2D_2$ or equivalent to these species. In quantifying the products of nitrogenase reduction of C_2D_2, Han and Newton[3] report that the molar absorptivity of the C_2H_3D peak is half of that of the C_2H_4 peak so they doubled the C_2H_3D peak height for their analysis. In similar measurements, Benton et al.[4] and Fisher et al.[5] report that the molar absorptivity of the *trans*-$C_2H_2D_2$ peak is half of that of the C_2H_4 and *cis*-$C_2H_2D_2$ peaks, but they don't explicitly address the molar absorptivity of the peak from the singly deuterated species, C_2H_3D, at 943 cm^{-1}. Due to this uncertainty in the literature, we performed a standard curve with C_2H_4 and C_2H_3D to

determine their molar absorptivities: C_2H_3D was quantitatively transferred to a 1 L Schlenk flask to a final pressure of 1 atm and a separate flask was purged with C_2H_4 for 10 minutes and vented to 1 atm. FTIR spectra were obtained using the same procedure that was used when analyzing the headspace of nitrogenase acetylene reduction assays except known quantities (50 – 100 µL) of 1 atm C_2H_4/C_2H_3D were transferred to the degassed sample chamber and FTIR spectra were obtained. The peak heights of the C_2H_4 (949 cm^{-1}) and C_2H_3D (943 cm^{-1}) stretches were plotted against the moles of these species in the sample chamber to obtain a standard curve. The absorbance from these stretches was calculated from the percent transmittance and this was plotted against the known amount of moles of the ethylene isotopologue in the FTIR gas cell (Figure S4). Trendlines were generated in Excel and the slope was the molar absorptivity of the species. In agreement with the report by Han and Newton, the molar absorptivity of C_2H_3D was about half of that of C_2H_4. We also simulated the molecular spectra of C_2H_4, C_2H_3D, and $C_2H_2D_2$ using TheoReTS[6] and obtained similar values for the molar absorptivities: 46.7 cm^{-1} for $C_2H_2D_2$, 24.3 cm^{-1} for C_2H_3D, and 41.7 cm^{-1} for C_2H_4. To our knowledge, this is the only reported standard curve of C_2H_4 and C_2H_3D by FTIR and is necessary for quantifying ethylene isotopologues by FTIR.

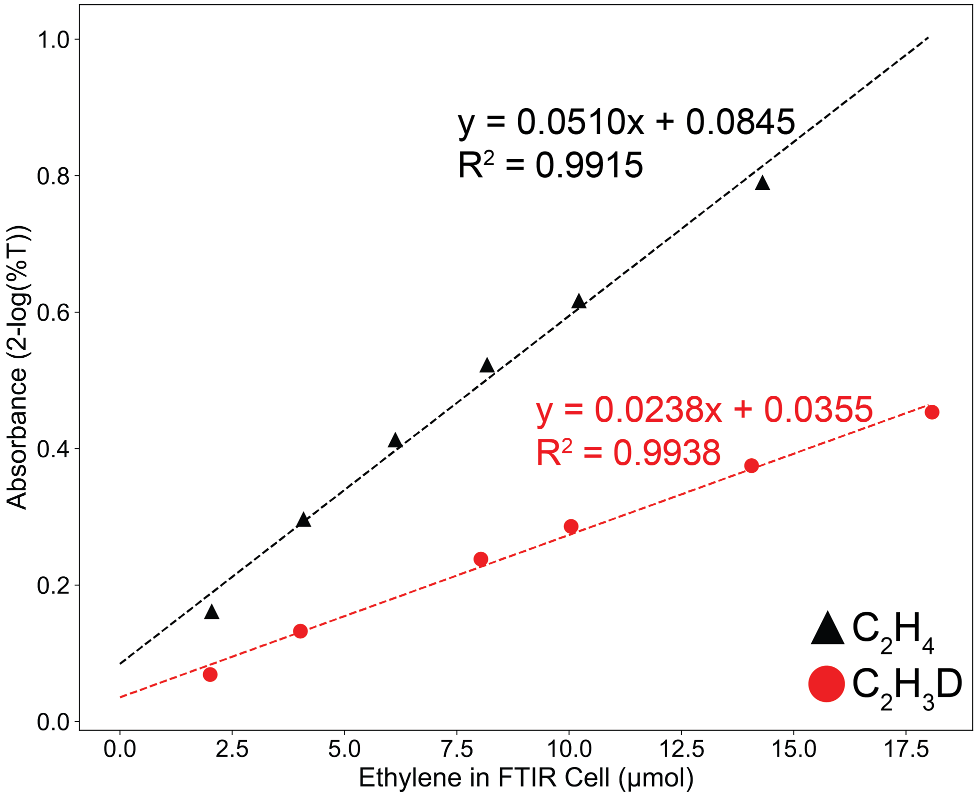

Figure S4: FTIR standard curves of ethylene isotopologues C_2H_4 and C_2H_3D. Peak transmittance values of the C_2H_4 stretch at 949 cm^{-1} (black) and the C_2H_3D stretch at 943 cm^{-1} (red) were converted to absorbance (Abs = 2-log(% Transmittance)) and plotted against the amount of ethylene present in the FTIR gas cell (µmol). Linear least squares fit trendlines were fit to the data in Excel and the slope of the line was the molar absorptivity of the species.

FTIR Spectra of Headspace of Acetylene Reduction Assays

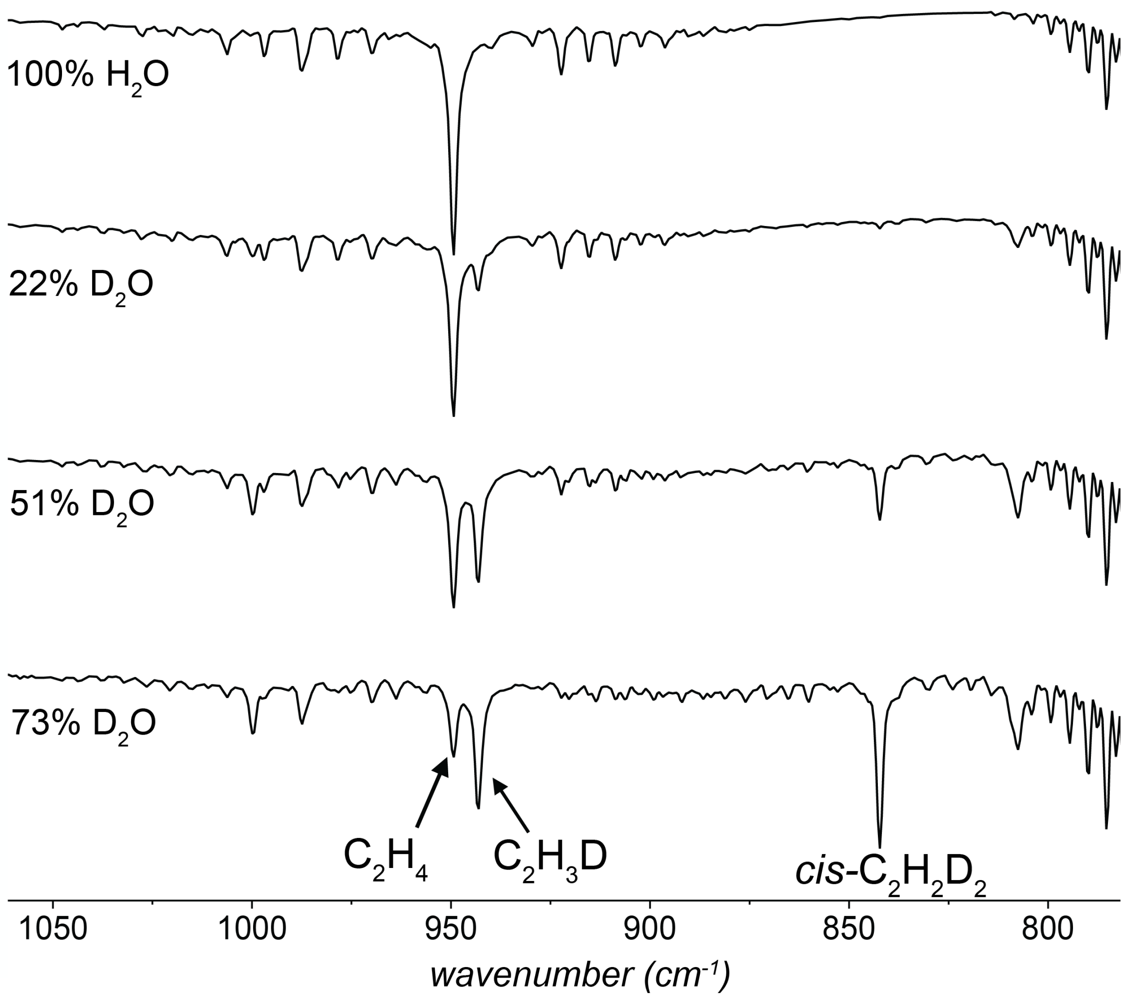

Figure S5: FTIR spectra of the headspace of acetylene reduction assays performed in varying mole fration of deuterium in the solvent. In 100% H_2O, the only observed stretch is at 949 cm^{-1} due to the presence of C_2H_4. Rotational stretches are observed on either side of the main C_2H_4 peak. In 22% D_2O, a peak emerges at 943 cm^{-1} due to C_2H_3D in the headspace. At 51% D_2O, the C_2H_3D peak at 943 cm^{-1} increases and a peak emerges at 843 cm^{-1} due to the presence of *cis*-$C_2H_2D_2$.

Table S2: Relative Amounts of Ethylene Isotopologue Products of Acetylene Reduction Assays Quantified by FTIR

Deuterium Enrichment	C_2H_4	C_2H_3D	$C_2H_2D_2$
22%*	54.9%	41.7%	3.4%
51%	31 ± 1%	54 ± 1%	14 ± 2%
73%	16 ± 1%	53.0 ± 0.9%	30.9 ± 0.7%

* only one trial performed

^{1}H NMR Spectra of Headspace of Proton Reduction Assays

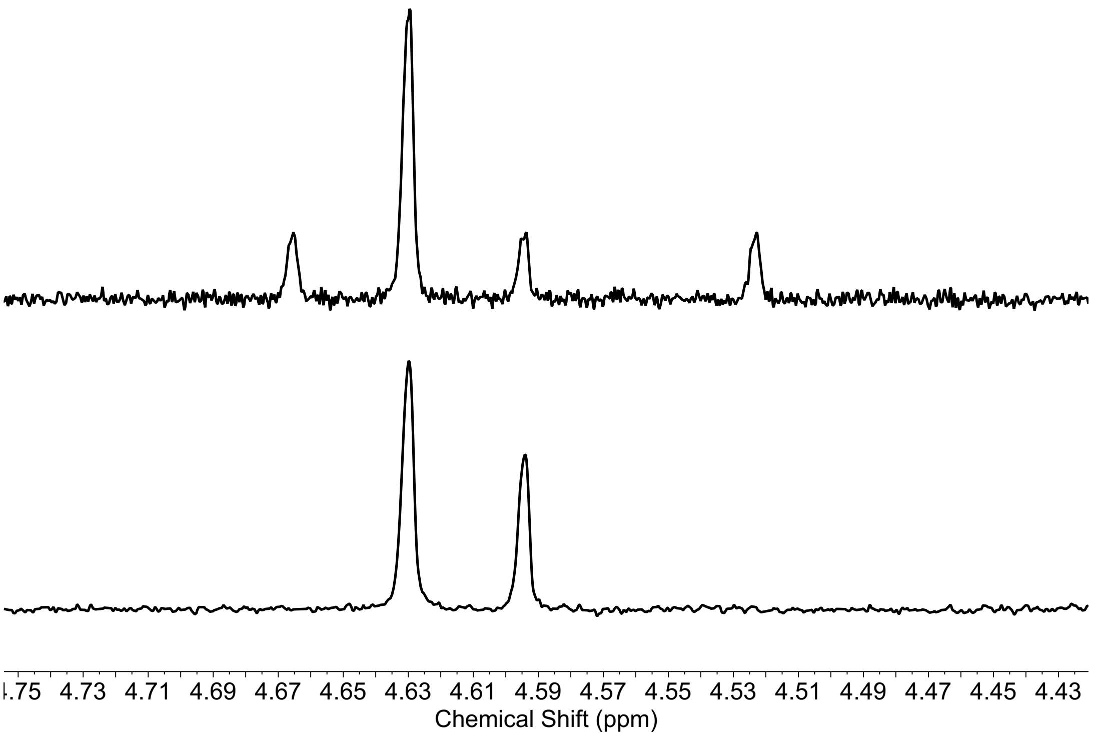

Figure S6: H_2/HD region of ^{1}H NMR spectra from headspace of proton reduction assay. Without deuterium decoupling (top), HD signal is a 1:1:1 triplet. Deuterium decoupling (bottom) condenses triplet into single peak. NMR tube filled with 1 mL $CDCl_3$ and capped with PTFE septa. Headspace from proton reduction assay (3 mL) transferred to a NMR tube with bubbling though $CDCl_3$. NMR performed on a Varian 600 MHz Spectrometer with 5 mm triple resonance inverse probe (parameters: 512 scans, 16 sec relaxation delay, pulse angle 90 °C). ^{1}H NMR ($CDCl_3$, 600 MHz) H_2: δ ppm 4.63 (s, 2H); HD: δ ppm 4.59 (t, J=48 Hz, 1H)

Note: We first attempted to quantify the products of proton reduction assays (H_2, HD, and D_2) in H_2O/D_2O with GC-MS. Modifications to the GC-MS system were made to optimize quantification of the hydrogen isotopologues: installation of 5 Å molecular sieve column for sufficient separation of H_2 from background argon; installation of external power supply to manually decrease the ionization energy to a level below that of the carrier gas,

He, which would interfere with the D_2 signal (both produce m/z = 4 ions). Despite these optimizations, GC-MS was not suitable for our system because of the considerable mass bias among D_2, HD, and H_2: the error in the standard curve of D_2 was a significant portion of the H_2 signal. When the headspace of nitrogenase proton reduction assays was analyzed with these GC-MS conditions, the results agreed with the quantification by ^{1}H NMR reported below; however, the precison was not satisfactory.

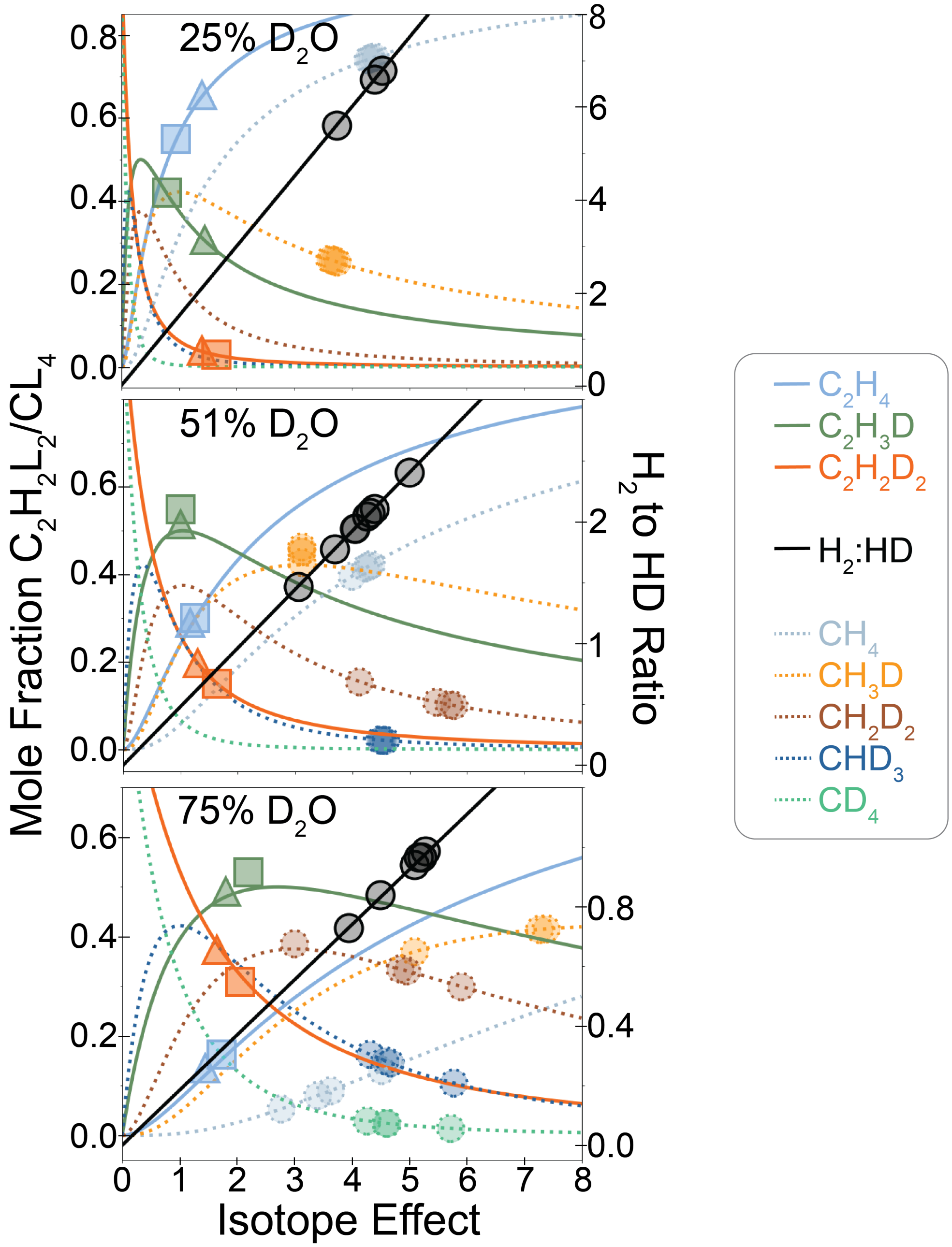
25% D2O
51% D2O
75% D2O
Mole Fraction C2H2L2/CL4
H2 to HD Ratio
Isotope Effect
C2H4
C2H3D
C2H2D2
H2:HD
CH4
CH3D
CH2D2
CHD3
CD4

Figure S7: Proton inventory analysis of acetylene, cyanide, and proton reduction by nitrogenase. Results of all assays at 25 % D_2O (top), 51% D_2O (middle) and 75% D_2O (bottom) are shown as markers. Solid lines are plots of Equations 1 - 3 (left axis; blue, green, and orange, respectively) and Equation 4 (right axis; black), the model expressions for the mole fraction of ethylene isotopologue product or H_2 to HD ratio as a function of the isotope effect. The dashed lines are plots of Equations (left axis; light blue, orange, brown, dark blue, teal, respectively), the model expressions for the mole fraction of methane isotopologue product as a function of the isotope efect. Markers are experimental data from the reduction assays with triangles as the results of GC-MS quantification of acetylene reduction assays, squares as the FTIR quantification of acetylene reduction assays, dotted circles as the GC-MS quantification of cyanide reduction assays and circles as the ^{1}H NMR quantification of proton reduction assays. The black circles are the experimental data from each proton reduction assay trial. All data plotted for cyanide and proton reduction data. Mean values plotted for acetylene reduction data and error bars are smaller than marker size.

Derivation of Isotope Effect Model Curves

The reduction of acetylene to ethylene and protons to H_2 involves the incorporation of 2 H/D atoms into the product. With each H/D addition, there is a probability, p, that a hydrogen atom is added and a probability, q, that a deuterium atom is added. The probability that a hydrogen is added is a function of the rate of this transfer and the mole fraction of hydrogen in the solution:[7]

$$p = \frac{k_H f_H}{k_H f_H + k_D f_D}$$

where k_H and k_D are the rates of hydrogen and deuterium addition, respectively, and f_H and f_D are the mole fractions of hydrogen and deuterium in the solvent, respectively. The isotope effect (IE) is defined as $\frac{k_H}{k_D}$ and the expression for p can be rewritten as:

$$p = \frac{IEf_H}{IEf_H + f_D}$$

It follows that the probability of adding a deuterium (instead of a hydrogen) can be wrtten as:

$$q = 1 - p = \frac{f_D}{IEf_H + f_D}$$

To generate H_2 and C_2H_4, two hydrogens are added. For HD and C_2H_3D, one hydrogen and one deuterium are added; and for D_2 and $C_2H_2D_2$, two deuteriums are added. Making the assumption that p and q are the same for the first and second proton addition, the mole fraction of dihydrogen and ethylene isotopologue products can be written as the product of the appropriate probabilities of adding H vs. D:

$$X_{C_2H_4} = X_{H_2} = p^2 = \frac{IE^2 f_H^2}{(IEf_H + f_D)^2} \tag{1}$$

$$X_{C_2H_3D} = X_{HD} = 2pq = \frac{2IEf_D f_H}{(IEf_H + f_D)^2} \tag{2}$$

$$X_{C_2H_2D_2} = X_{HD} = q^2 = \frac{f_D^2}{(IEf_H + f_D)^2} \tag{3}$$

For the results of the proton reduction assays, only the ratio of H_2 to HD could be obtained so the isotope effect was calculated by the following equation:

$$\frac{X_{H_2}}{X_{HD}} = \frac{IE^2 f_H^2}{(IEf_H + f_D)^2} \cdot \frac{(IEf_H + f_D)^2}{2IEf_D f_H} = \frac{IEf_H}{2f_D} \tag{4}$$

In order to explicitly calculate the isotope effect from the relative amounts of isotopologue products, equations 1-4 were solved for the isotope effect:[8]

$$IE_{C_2H_4} = \frac{f_D\sqrt{X_{C_2H_4}}}{f_H - f_H\sqrt{X_{C_2H_4}}} \tag{5}$$

$$IE_{C_2H_3D} = \frac{\sqrt{-f_D^2 f_H^2(2X_{C_2H_3D} - 1)} - X_{C_2H_3D} f_D f_H + f_D f_H}{f_H^2 X_{C_2H_3D}} \tag{6}$$

$$IE_{C_2H_2D_2} = \frac{f_D\left(\frac{1}{\sqrt{X_{C_2H_2D_2}}} - 1\right)}{f_H} \tag{7}$$

$$IE_{H_2/HD} = \frac{2f_D}{f_H} \cdot \frac{X_{H_2}}{X_{HD}} \tag{8}$$

Least Square Fit of Acetylene and Proton Reduction Assay Results

A least squares fit was performed on the acetylene reduction data across all levels of deuterium enrichment for each ethylene isotopologue (Figure S8). The relative amount of each ethylene isotopologue (percentage of total ethylene produced) was plotted against the mole fraction of deuterium in the solvent. Then, the scipy.optimize.curve_fit function was used in Jupyter notebooks to perform a non-linear least squares fit of the data to Equations 1-3. The isotope effects obtained from these fits are 1.28 ± 0.04, 1.4 ± 0.1, and 1.59 ± 0.05 for C_2H_4, C_2H_3D, and $C_2H_2D_2$, respectively.

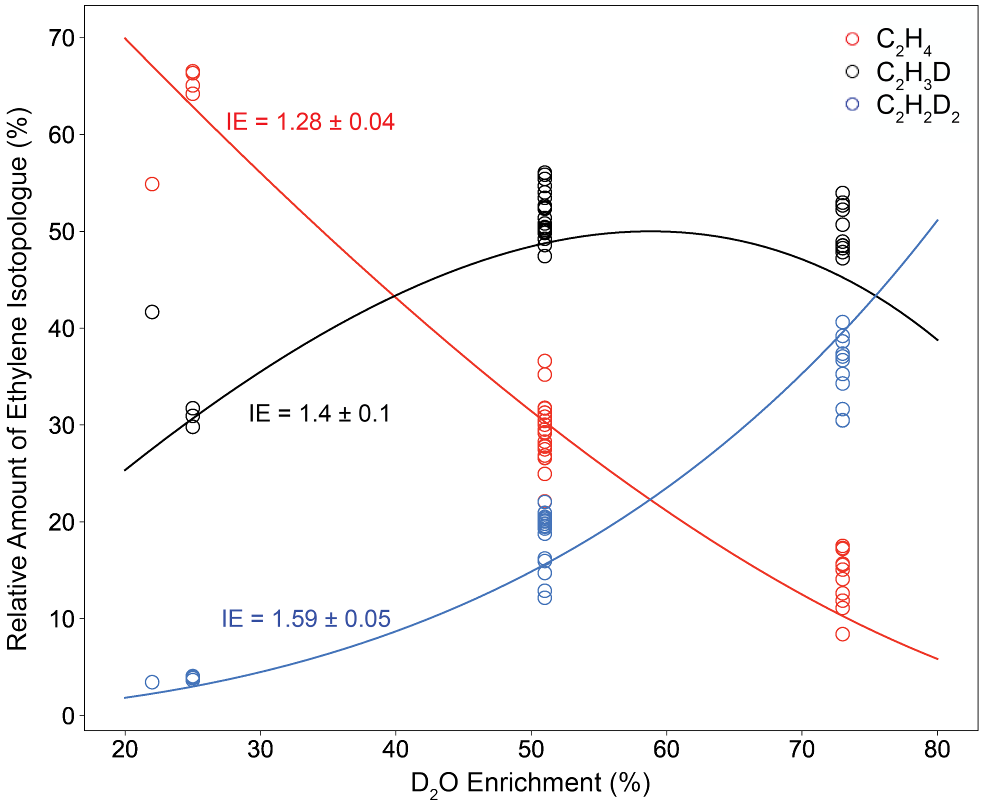

Figure S8: Least squares fits (solid lines) of Equations 1-3 to experimental acetylene reduction assay results (points). Relative amounts of C_2H_4(red), C_2H_3D(black), and $C_2H_2D_2$(blue) plotted against the percentage of deuterium enrichment in the buffer. Plots and least squares fit (scipy.optimize.curve_fit) generated in Jupyter Notebooks.

The results of the proton reduction assay were fit to Equation 7 using the same method as described above (Figure S9). The isotope effect value obtained from this fit was 4.2 ± 0.1.

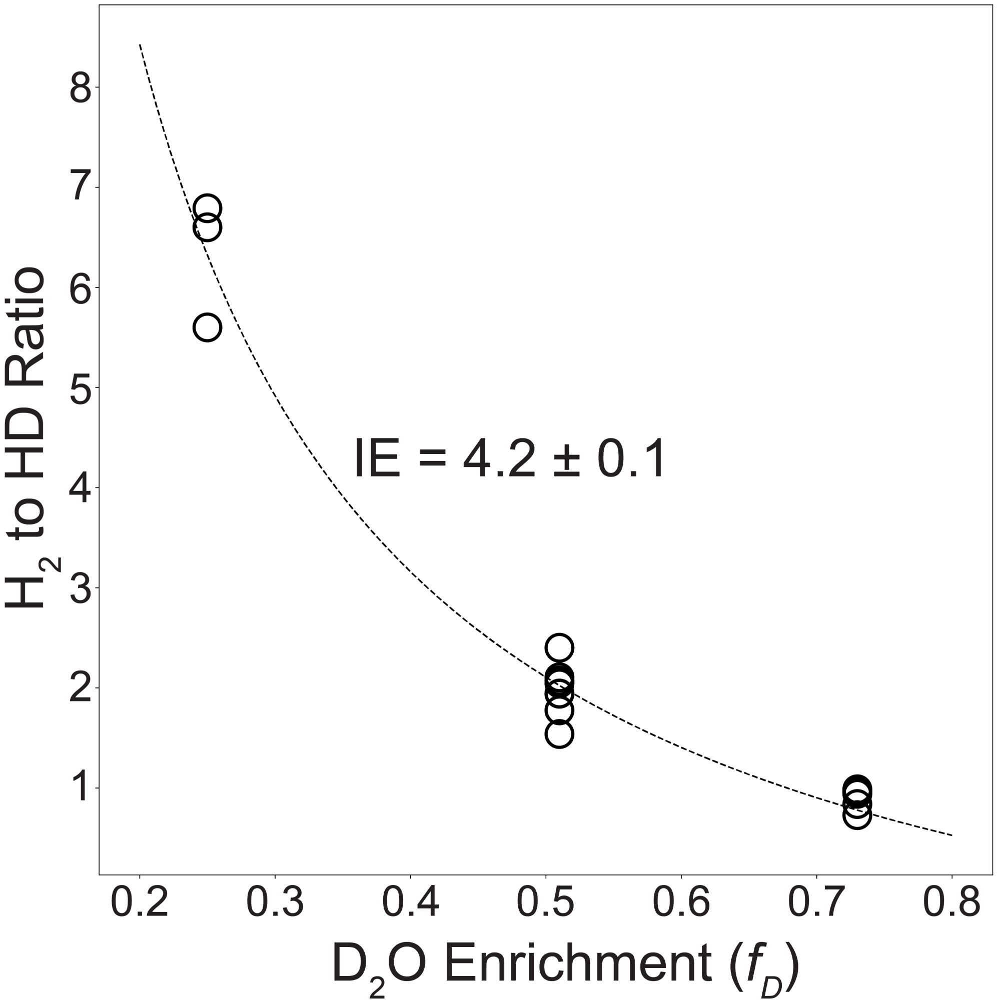

Figure S9: Least squares fit of experimental proton reduction assay results to Equation 7. Ratio of H_2 to HD plotted against the percentage of deuterium enrichment in the buffer. Experimental data are black circles and least squares fit is dotted black line. Plots and least squares fit (scipy.optimize.curve_fit) generated in Jupyter Notebooks.

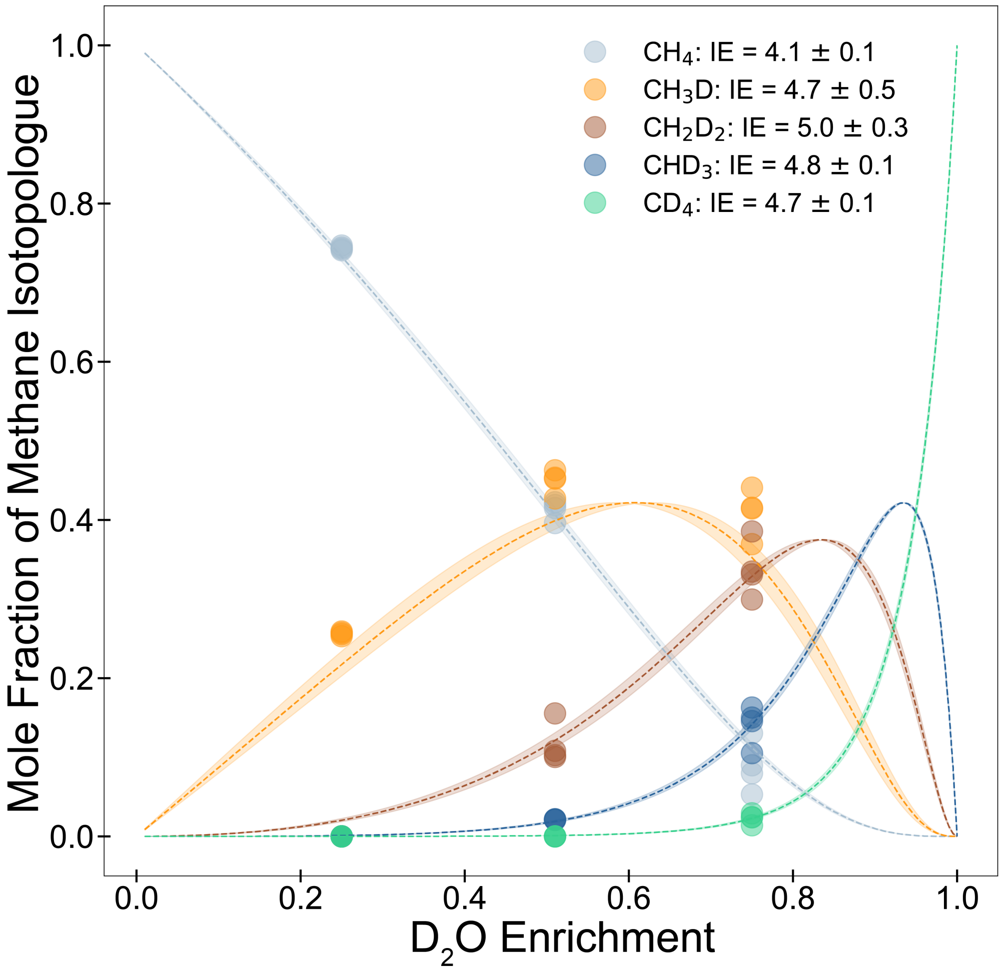

Figure S10: Least squares fits (dashed lines) of Equations to experimental cyanide reduction assay results (points). Mole fraction of CH_4 (light blue), CH_3D (orange), CH_2D_2 (brown), CHD_3 (dark blue), CD_4 (teal), plotted against the mole fraction of deuterium enrichment in the buffer. Plots and least squares fit (scipy.optimize.curve_fit) generated in Jupyter Notebooks.

Estimation of Fractionation Factor of H_2 Production by Nitrogenase

The level of incorporation of heavy isotopes into enzyme products are also of interest in the field of geochemistry and these results are termed *fractionation factors* instead of isotope effects. The fractionation of a heavy isotope such as deuterium is related to the ratio of the amount of deuterium in the product (in parts per million) to the natural abundance of deuterium in a standard water sample. Mechanistic conclusions are rarely made from these fractionation factors and instead, they provide clues as to the microbial sources of important chemicals such as NH_3 and CH_4 in the environment.[9–13] Upon inspection of the definitions of isotope effect and fractionation factor, in the case of solvent isotope effect experiments where protons are sourced from the solvent as in reduction by nitrogenase, the fractionation factor is the reciprocal of the isotope effect. In this work, we calculate isotope effects of nitrogenase reduction assays; however, the results can also easily be expressed as fractionation factors and are relevant to studies of geological nitrogen fixation. In reports measuring deuterium fractionation during dihydrogen production by cyanobacteria[14] or purified hydrogenases[10] significant depletion of deuterium was also observed. When we convert our isotope effect values to fractionation factors (Table S3) our results are comparable to these studies on hydrogenases, which could indicate that deuterium depletion is a common feature of H_2 production. In fact, these levels of deuterium fractionation are similar to that of H_2 production by the electrolysis of water.[14]

The fractionation factor (α) is defined as the ratio of deuterium to hydrogen in the products divided by the ratio of deuterium to hydrogen in the solvent:

$$\alpha_{H_2 evolution} = \frac{R_{H_2}}{R_{H_2O}} \tag{9}$$

where R_{H_2} is the ratio of D to H in the L_2 products and R_{H_2O} is the ratio of D to H in the

solvent (L is the nomenclature for either H or D). In order to calculate the D to H ratio of the L_2, we need to know the relative amount of D_2 produced, which we weren't able to quantify by ^{1}H NMR. However, we can calculate an estimate of the relative amounts of H_2, HD and D_2 using the isotope effect that we calculated from equation 5, by inserting the calculated isotope effect into equations 1-3. These calculations were performed with our proton reduction data and the results are shown in Table S3. The fractionation factor was estimated to be 0.24 $\pm$ 0.03, 0.25 $\pm$ 0.05, and 0.20 $\pm$ 0.02 from assays performed in 25% D_2O, 51% D_2O, and 73% D_2O, respectively. All of the values are within error of each other.

Table S3: Calculating Fractionation Factor of Proton Reduction

sample	H2/HD	IE	calc_H_2	calc_HD	calc_D_2	R_H_2	α
**25% D_2O trial 1**	6.790778	4.527186	0.867544	0.127753	0.004703	0.073629	0.220888
**25% D_2O trial 2**	5.600473	3.733649	0.842796	0.150487	0.006718	0.089278	0.267835
**25% D_2O trial 3**	6.600526	4.400351	0.864124	0.130917	0.004959	0.075752	0.227255
**average**	6.330593	4.220395	0.858154	0.136386	0.005460	0.079553	**0.238659**
**stdev**	0.639417	0.426278	0.013410	0.012314	0.001097	0.008489	**0.025467**

**51% D_2O trial 1**	2.105380	4.382628	0.653009	0.310162	0.036830	0.237487	0.228174
**51% D_2O trial 2**	2.044485	4.255867	0.645607	0.315780	0.038614	0.244560	0.234970
**51% D_2O trial 3**	1.942120	4.042781	0.632438	0.325643	0.041919	0.257451	0.247355
**51% D_2O trial 4**	2.400800	4.997584	0.684978	0.285312	0.029710	0.208264	0.200097
**51% D_2O trial 5**	1.539298	3.204253	0.569750	0.370136	0.060114	0.324823	0.312085
**51% D_2O trial 6**	1.775673	3.696300	0.608844	0.342881	0.048275	0.281583	0.270541
**51% D_2O trial 7**	2.071615	4.312341	0.648942	0.313254	0.037803	0.241358	0.231893
**51% D_2O trial 8**	1.943430	4.045508	0.632613	0.325514	0.041874	0.257277	0.247188
**average**	1.977850	4.117158	0.634523	0.323585	0.041892	0.256600	**0.246538**
**stdev**	0.251864	0.524288	0.033977	0.024957	0.009058	0.034556	**0.033201**

**73% D_2O trial 1**	0.986996	5.283329	0.437567	0.447843	0.114590	0.511742	0.189275
**73% D_2O trial 2**	0.965335	5.167379	0.430994	0.451014	0.117991	0.523225	0.193522
**73% D_2O trial 3**	0.839624	4.494459	0.389863	0.469054	0.141083	0.601564	0.222496
**73% D_2O trial 4**	0.728799	3.940912	0.351766	0.482665	0.165569	0.686060	0.253748
**73% D_2O trial 5**	0.965839	5.222683	0.434147	0.449503	0.116350	0.517685	0.191472
**73% D_2O trial 6**	0.942058	5.094090	0.426766	0.453015	0.120219	0.530753	0.196306
**average**	0.904775	4.867142	0.411850	0.458849	0.129300	0.561838	**0.207803**
**stdev**	0.100756	0.536295	0.034179	0.013966	0.020242	0.069175	**0.025585**

Time Course of NaCN reduction assays in H_2O and D_2O

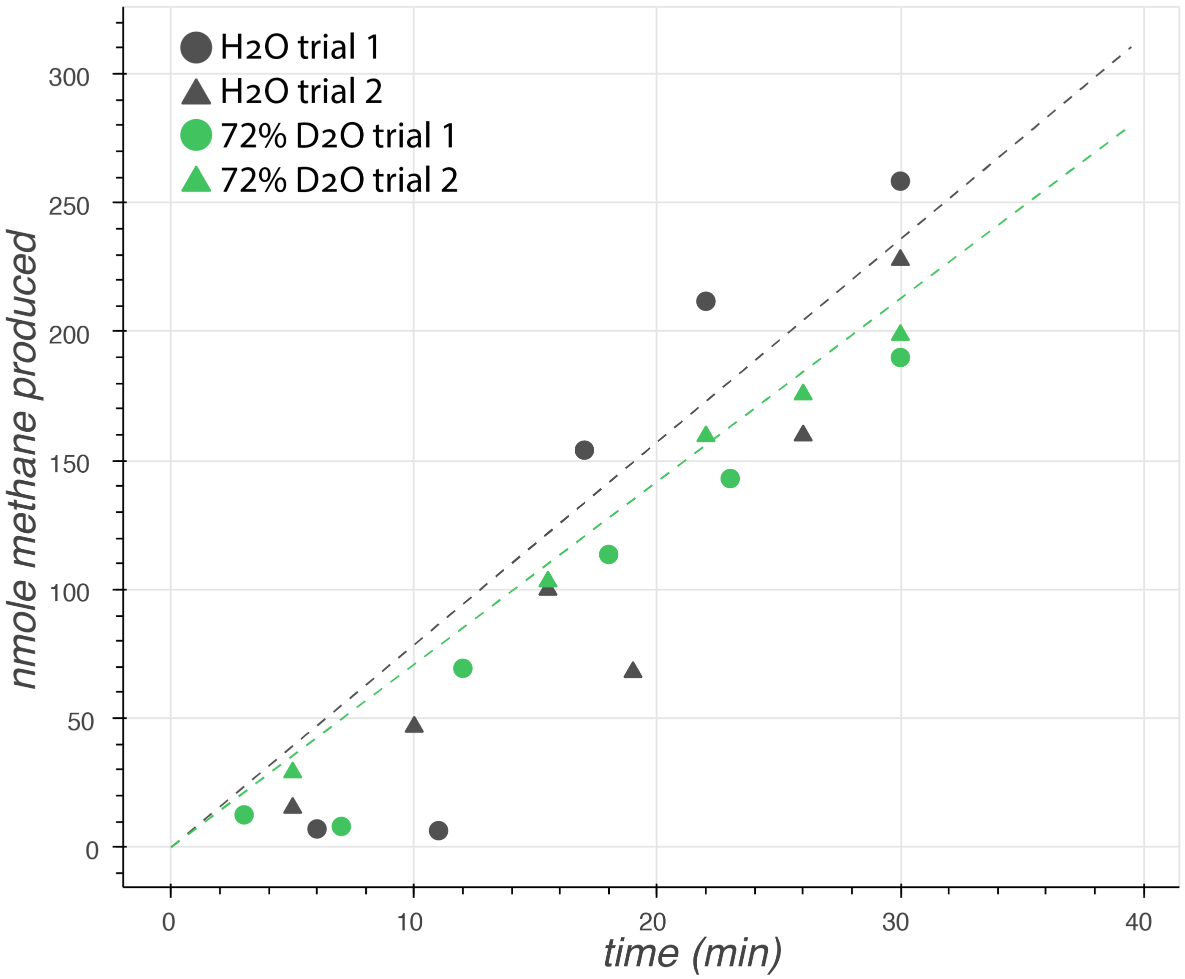

Figure S11: Time course of NaCN reduction assays in H_2O (grey) and 72% D_2O (green). Dashed lines are linear least squares fit to trial 2 from assays performed in H_2O (grey dashed, rate = 7.861 nmol methane produced min^{-1}) or 72% D_2O (green, rate = 7.095 nmol methane produced min^{-1}).

Mass Spectra of Methane Peak of Headpsace of NaCN Reduction Assays

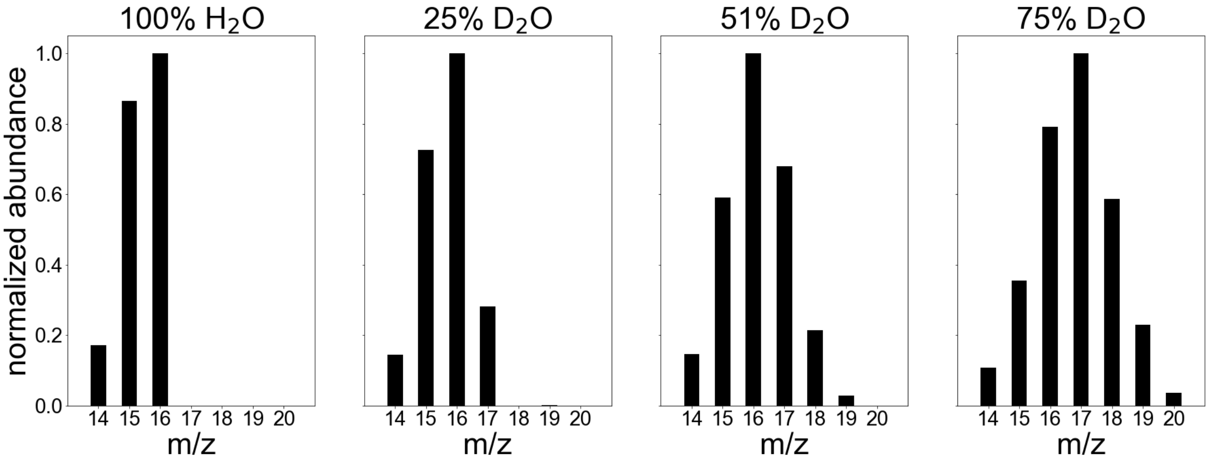

Figure S12: Mass spectra of methane peaks of headspace of acetylene reduction assays in 100% H_2O, 25% D_2O, 51% D_2O, and 75% D_2O. Abundance normalized to peak of greatest abundance. Mass spectra generated by integrating under methane peak of extracted ion chromatograms of m/z = 14-20. As mole fraction of D_2O in buffer increases, greater abundance seen at m/z = 17, m/z = 18, m/z = 19, and m/z = 20, corresponding to increase in CH_3D, CH_2D_2, CHD_3 and CD_4 in the headspace.

Table S4: Relative Amounts of Methane Isotopologue Products of Cyanide and Methyl Isocyanide Reduction Assays Quantified by GC-MS

NaCN Reduction					
	**CH_4**	**CH_3D**	**CH_2D_2**	**CHD_3**	**CD_4**
**25% D_2O**	74 ± 0.3	25 ± 0.3	0	0	0
**51% D_2O**	41 ± 1	45 ± 2	12 ± 3	2.13 ± 0.04	0
**75% D_2O**	9 ± 3	41 ± 3	34 ± 4	14 ± 2	2.3 ± 0.6
CH_3NC Reduction					
**51% D_2O**	37 ± 1	45 ± 2	14 ± 2	2.6 ± 0.1	0

References

(1) Yang, Z. Y.; Khadka, N.; Lukoyanov, D.; Hoffman, B. M.; Dean, D. R.; Seefeldt, L. C. On reversible H2 loss upon N2 binding to FeMo-cofactor of nitrogenase. *PNAS USA* **2013**, *110*, 16327–16332.

(2) Lin-Vien, D.; Fateley, W. G.; Davis, L. C. Estimation of Nitrogenase Activity in the Presence of Ethylene Biosynthesis by Use of Deuterated Acetylene as a Substrate. *App. Environ. Microbiol.* **1989**, *55*, 354–359.

(3) Han, J.; Newton, W. E. Differentiation of Acetylene-Reduction Sites by Stereoselective Proton Addition during *Azotobacter Vinelandii* Nitrogenase-Catalyzed C_2D_2 Reduction. *Biochemistry* **2004**, *43*, 2947–2956.

(4) Benton, P. M.; Christiansen, J.; Dean, D. R.; Seefeldt, L. C. Stereospecificity of Acetylene Reduction Catalyzed by Nitrogenase. *J. Am. Chem. Soc.* **2001**, *123*, 1822–1827.

(5) Fisher, K.; Dilworth, M. J.; Kim, C. H.; Newton, W. E. *Azotobacter vinelandii* Nitrogenases with Substitutions in the FeMo-Cofactor Environment of the MoFe Protein: Effects of Acetylene or Ethylene on Interactions with H^+, HCN, and CN^-. *Biochemistry* **2000**, *39*, 10855–10865.

(6) Viglaska, D.; Rey, M.; Delahaye, T.; Nikitin, A. V. First-principles calculations of infrared spectra for three ethylene isotopologues: 13C2H4, 13C12CH4 and 12C2H3D. *J. Quant. Spectrosc. Radiat. Transf.* **2019**, *230*, 142–154.

(7) Spencer, R. W.; Daniels, L.; Fulton, G.; Orme-Johnson, W. H. Product Isotope Effects on *in vivo* Methanogenesis by *Methanobacterium thermoautotrophicum*. *Biochemistry* **1980**, *19*, 3678–3683.

(8) Khushvakov, J.; Nussbaum, R.; Cadoux, C.; Duan, J.; Stripp, S. T.; Milton, R. D. Fol-

lowing Electroenzymatic Hydrogen Production by Rotating Ring-Disk Electrochemistry and Mass Spectrometry. *Angew. Chem. Int. Ed.* **2021**, *60*, 10001–10006.

(9) Luxem, K. E.; Leavitt, W. D.; Zhang, X. Large Hydrogen Isotope Fractionation Distinguishes Nitrogenase-Derived Methane from Other Methane Sources. *Appl. Environ. Microbiol* **2020**, *86*, e00849–20.

(10) Yang, H.; Gandhi, H.; Cornish, A. J.; Moran, J. J.; Kreuzer, H. W.; Ostrom, N. E.; Hegg, E. L. Isotopic Fractionation Associated with [NiFe]- and [FeFe]-hydrogenases. *Rapid Communications in Mass Spectrometry* **2016**, *30*, 285–292.

(11) Löffler, M.; Kümmel, S.; Vogt, C.; Richnow, H. H. H2 kinetic isotope fractionation superimposed by equilibrium isotope fractionation during hydrogenase activity of D. Vulgaris Strain Miyazaki. *Front. Microbiol.* **2019**, *10*.

(12) Yang, H.; Gandhi, H.; Shi, L.; Kreuzer, H. W.; Ostrom, N. E.; Hegg, E. L. Using gas chromatography/isotope ratio mass spectrometry to determine the fractionation factor for H 2 production by hydrogenases. *Rapid Commun. Mass Spectrom.* **2012**, *26*, 61–68.

(13) Zhang, X.; McRose, D. L.; Darnajoux, R.; Bellenger, J. P.; Morel, F. M.; Kraepiel, A. M. Alternative nitrogenase activity in the environment and nitrogen cycle implications. *Biogeochemistry* **2016**, *127*, 189–198.

(14) Luo, Y.-H.; Sternberg, L.; Suda, S.; Kumazawa, S.; Mitsui, A. Extremely Low D/H Ratios of Photoproduced Hydrogen by Cyanobacteria. *Plant Cell Physiol.* **1991**, *32*, 897–900.
